# Epigenome Engineering Using dCas Systems for Biomedical Applications and Biotechnology: Current Achievements, Opportunities and Challenges

**DOI:** 10.3390/ijms26136371

**Published:** 2025-07-02

**Authors:** Maxim A. Kovalev, Naida Yu. Mamaeva, Nikolay V. Kristovskiy, Pavel G. Feskin, Renat S. Vinnikov, Pavel D. Oleinikov, Anastasiia O. Sosnovtseva, Valeriy A. Yakovlev, Grigory S. Glukhov, Alexey K. Shaytan

**Affiliations:** 1 Department of Biology, Lomonosov Moscow State University, 119234 Moscow, Russia; 2 Department of Fundamental Physical and Chemical Engineering, Lomonosov Moscow State University, 119234 Moscow, Russia; 3 Center for Precision Genome Editing and Genetic Technologies for Biomedicine, Engelhardt Institute of Molecular Biology, Russian Academy of Sciences, 119991 Moscow, Russia; 4 Faculty of Biology, MSU-BIT Shenzhen University, Shenzhen 518172, China; 5 Institute of Gene Biology, 119334 Moscow, Russia

**Keywords:** epigenetics, epigenome editing, epigenome engineering, epigenetic engineering, CRISPR/Cas, dCas, biomedical applications, biotechnology

## Abstract

Epigenome engineering, particularly utilizing CRISPR/dCas-based systems, is a powerful strategy to modulate gene expression and genome functioning without altering the DNA sequence. In this review we summarized current achievements and prospects in dCas-mediated epigenome editing, primarily focusing on its applications in biomedicine, but also providing a wider context for its applications in biotechnology. The diversity of CRISPR/dCas architectures is outlined, recent innovations in the design of epigenetic editors and delivery methods are highlighted, and the therapeutic potential across a wide range of diseases, including hereditary, neurodegenerative, and metabolic disorders, is examined. Opportunities for the application of dCas-based tools in animal, agricultural, and industrial biotechnology are also discussed. Despite substantial progress, challenges, such as delivery efficiency, specificity, stability of induced epigenetic modifications, and clinical translation, are emphasized. Future directions aimed at enhancing the efficacy, safety, and practical applicability of epigenome engineering technologies are proposed.

## 1. Introduction

Fundamental advances in genome editing and genetic engineering technologies are currently shaping progress in life sciences, including practical applications in biomedicine and biotechnologies [1]. While altering the DNA sequence of the genome is a powerful way to achieve certain goals, technologies that control and modify the way in which the genome functions (even without altering its sequence) open up many more opportunities for developing new therapeutic modalities or engineering new organisms for biotechnological needs. Genome regulation and control in eukaryotic organisms are heavily dependent on epigenetic mechanisms [2,3]. Selectively activating or repressing such mechanisms in a locus-specific manner constitutes the idea of **epigenome editing**. This can be achieved using engineered biomolecular tools that combine **sequence-specific DNA-binding domains (such as polydactyl zinc-finger proteins or dCas-based complexes) with various epigenetic effector domains** (discussed below). Akin to genome engineering, **epigenome engineering** is a broader term comprising the development and use of various molecular tools and genetic constructs to confer new functional properties to cells or organisms by employing epigenetic mechanisms.

According to the current understanding, epigenetic regulation in eukaryotic cells is achieved through local chemical, conformational, and compositional changes in the chromatin structure and specialized molecular machinery that introduces and senses such changes [3]. Chemical modifications comprise DNA methylation and post-translational modifications of proteins associated with the DNA—first and foremost, histones, which tightly associate with DNA to form nucleosomes [4]. The proteins involved in these modifications are usually classified into three groups: “writers”, “readers”, and “erasers”. 5-methylcytosine (5mC) is a common DNA modification found in the CpG islands near promoter regions of certain genes (mainly, retrotransposons, imprinted genes and also on the inactivated X-chromosome) where it leads to a decrease in the activity of the respective part of the genome [5,6]. “Writers” of such tags are DNA methyltransferases (in humans these are five genes, namely, *DNMT1*, *DNMT2*, *DNMT3A*, *DNMT3B* and *DNMT3L* [7]), and the 5mC removal process is initiated by TET dioxygenases, such as TET1, and then finished by thymine DNA glycosylase (TDG) enzyme as a part of the base excision repair (BER) pathway [8]. Acetylation of histones is one of the best-known modifications, which almost always has an activating effect [9]. Histone acetylation is carried out by histone acetyltransferases, such as CBP and p300, and the “erasers” are histone deacetylases including HDAC1, HDAC3, HDAC6, etc. [10]. Arginine and lysine residues are often methylated in histones. Unlike acetylation, the effects of histone methylation are highly dependent on the particular histone site at which it occurs [9]. For example, the H3K4me3 and H3K36me3 tags are often associated with actively transcribed regions, while H3K9me3 and H3K27me3 are well-known markers of heterochromatin. Methyl groups are placed on histones by methyltransferases (such as EZH2, PRDM9, etc.) and removed by demethylases (such as LSD1) [11]. Other histone modifications include phosphorylation, sumoylation, ubiquitination, and many others, including the recently discovered dopaminylation and serotonylation [9]. Histone tail cleavage is a potential modification [12]. In addition to such relatively simple modifications, there are other levels of epigenetic regulation. The repositioning of nucleosomes and incorporation of histone variants into them are important mechanisms accomplished by chromatin remodeling complexes whose activity is, in turn, tightly regulated [13,14]. Recruitment of chromatin proteins (H1 histones, HP1 protein, etc.) [15,16], lncRNAs [17], modification of 3D structures, including chromatin looping [18], and the formation of liquid–liquid phase separation [19], are other important mechanisms of epigenetic regulation.

Epigenetic markup of the genome and the corresponding epigenetic mechanisms are at the core of such processes as cellular differentiation, regulation of genome activity due to external factors and stimuli, transmission of epigenetic states through mitosis or even inter- and transgenerational inheritance [20]. In humans, epigenetic mechanisms are implicated in many disorders, such as cancer, autoimmune, cardiovascular, metabolic, neurodegenerative, psychiatric, and many other diseases [21], opening possibilities for therapeutic interventions by targeting these mechanisms. Aging, exposure to various environmental conditions and lifestyle factors also affect the cellular epigenome [22], suggesting targeted epigenetic editing may mimic or reverse the effects of such processes. In addition, controlling the epigenome of economically important plants, animals and other organisms opens up the widest biotechnological possibilities, which is especially relevant in the context of the growing world population and climate change. Moreover, since certain epigenetic modifications in plants are heritable there is a potential possibility for improving crops’ properties without altering their genomes, hence remaining in the legal regulatory landscape applicable to “non-GMO” organisms.

The course of development of the targeted epigenome editing tools followed closely that of genome editing tools, since both technologies require programmable DNA sequence-specific targeting domains. Engineered polydactyl zinc-finger (ZF) proteins were the first to provide such programmable functionality that was used to create synthetic transcription factors back in the 1990s [23]. However, zinc-finger-based DNA binders are not always easy to design [24]. An easier alternative based on TAL (transcription activator-like) effectors appeared more than a decade later and was used in 2011 by Feng Zhang and colleagues to create sequence-specific transcription regulators [25]. The discovery and subsequent repurposing of the CRISPR/Cas9 system revolutionized the field of genome editing starting from 2012 [26,27,28]. It was now possible to target a protein at a specific genome locus simply by designing a special RNA molecule (sgRNA) that had a spacer segment complementary to that locus. Already in 2013 it was shown that the catalytically inactive Cas9 nuclease (dead Cas9, or dCas9) fused with epigenetic effectors may be used to inhibit or activate transcription in eukaryotes in a locus-specific manner (termed CRISPRi and CRISPRa technologies, respectively) [29]. The RNA-based genome targeting mechanism in such systems allows for unprecedented relative simplicity and scalability of experimental studies. For instance, through the use of sgRNA libraries the so-called CRISPRa screens can be performed, where all genes can be screened to find out the upregulation of which one is sufficient to elicit a given phenotype [30]. The accessibility of dCas-based epigenome editors sparked an ever-growing flow of studies where new types of such editors were being developed and used to tackle fundamental and applied problems in medicine and biotechnology. A comparison of dCas-based epigenetic tools with genome-editing technologies—such as nucleases, base editors, and prime editors—highlighting their safety, reversibility, and delivery constraints, is provided in Supplementary Table S4.

In this review, we provide an overview of various dCas-based epigenomic editors and discuss the recent literature showing their potential in solving real-life problems, particularly tackling human diseases. To put these data into context an overview of prospective applications of epigenome engineering in animal, industrial and agrobiotechnologies is also given, as well as a discussion of the corresponding challenges and ways to address them. While epigenome engineering is currently a hot topic and a number of nice reviews and books have been published in recent years [31,32,33,34], the distinctive feature of this review is an in-depth analysis of the most recent literature concerning the practical applications of this technology.

## 2. The Diversity of CRISPR/Cas-Based Tools for Epigenome Engineering

**Figure 1:**
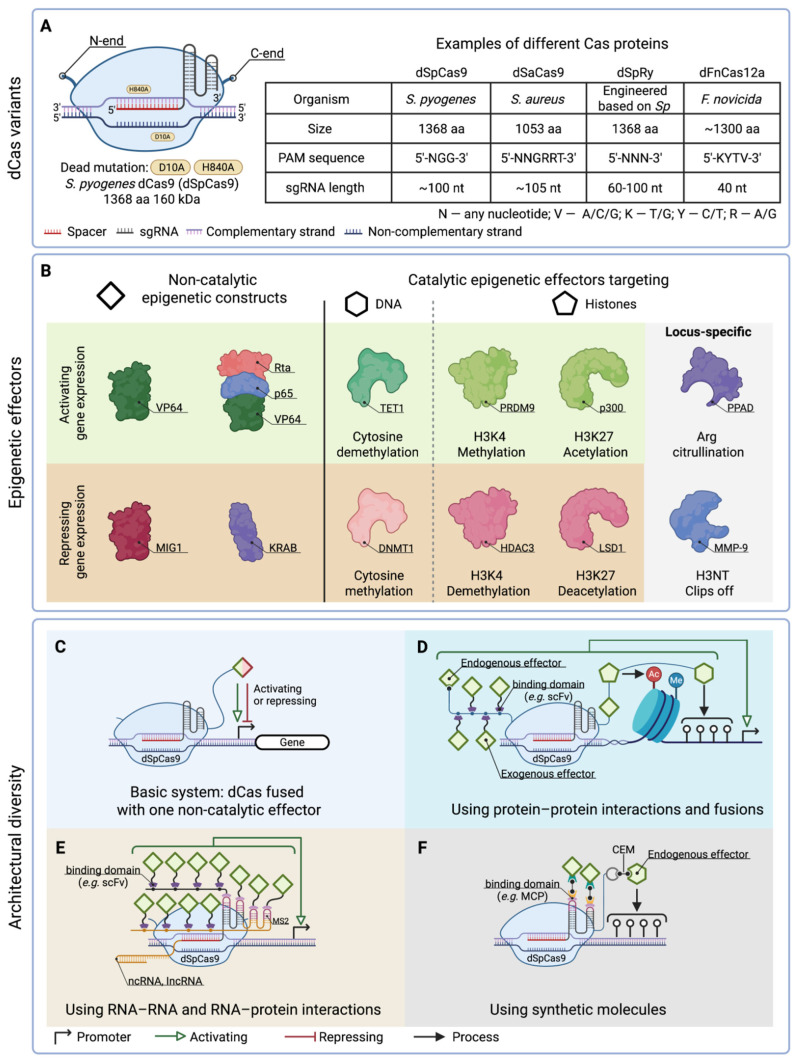
Basic representations of epigenetic system construction based on dCas proteins. (**A**) Schematic structure of SpdCas9 and key parameters of other popular dCas proteins. (**B**) Variety of effector domain types. (**C**) Example of a simple system based on SpdCas9 where the effector domain (activator or repressor) is attached to the C-terminal end of the Cas protein. (**D**) Variety of architectures based on modification of the C- and N-terminals of the Cas-protein. (**E**) Variety of architectures based on modifying sgRNA: MS2-gRNAs (gRNA 2.0); scRNA; Casilio; CRISPR-Display. (**F**) Variety of architectures based on chemical epigenetic modifiers (CEMs). Created with BioRender.com.

Over the last 10 years the family of designed CRISPR/dCas-based tools for epigenome editing/engineering has grown considerably and is continuing to expand. Although such tools have been reviewed previously [31,34,35], new, more efficient and flexible dCas-based systems continue to emerge. In this section, without unnecessary repetition, we summarize the variability of such systems highlighting recent advancements and future directions for their improvement. The variety of existing mechanisms and architectures for epigenome editing based on CRISPR systems are summarized in Figure 1. A representative list of studies that used different dCas-based epigenetic editors may be found in Supplementary Table S1, also available online as an interactive table at https://intbio.github.io/Kovalev_et_al_2025/ST1 (accessed on 24 June 2025).

The primary component of dCas-based epigenome editing tools is the catalytically inactive dCas protein itself, together with the RNA molecule that enables its targeting at the specific locus within the genome (Figure 1A). The specific binding of the dCas-sgRNA complex requires the presence of a short protospacer adjacent motif (PAM) (3–6 nucleotides). PAM is recognized by a specific protein domain which triggers a conformational change in Cas-protein accompanied by the melting of the DNA double helix that, in turn, facilitates the formation of an R-loop between an RNA spacer (approximately 20 nucleotides long) and the target DNA segment (protospacer) [36]. Likewise for genome editing, the catalytically inactive Cas9 protein from *Streptococcus pyogenes* (*Sp*dCas9) was the first to be widely applied for epigenome editing among members of the Cas-protein family. However, other dCas proteins are both orthologs of SpCas9 from different organisms (SaCas9 and CjCas9) [37], representatives of other types of dCas systems (e.g., Cas12a, Cas12b, Cas12f, CasΦ/Cas12j—which belong to class II type V where DNA target binding, cleavage and pre-crRNA processing functions are performed by a single protein [38]), and artificially engineered variants have since then been applied for epigenome editing (Figure 1A). Different dCas systems differ by their size (e.g., 1368 aa for SpdCas9, 422 aa for AsdCas12f), activity, recognizable PAM sequence, gRNA length and its modifications, and the ability to preprocess its RNA molecules (practically useful for encoding many guide-RNA molecules via a single array) [39]. The number of engineered Cas proteins and systems for gene editing is now enormous [40]. Only some of them have been adapted for epigenome editing (our literature analysis suggests that at least 15 different dCas proteins have been reported in epigenome editing applications—see Supplementary Table S1), and an even smaller fraction have been the focus of studies that systematically compare their activity. One study measured the efficiency of the activation and repression of gene transcription of various representatives of dCas proteins in human cell lines [41]. Although the activation/repression efficiency was different in each cell type, the dCas9 and dCas12a orthologs showed higher activities than other dCas proteins [42]. For therapeutic applications smaller dCas proteins are of great utility (e.g., dCas12f—the basis of the dCas-MINI system [42]), since conventional dCas9-based constructs hardly fit into the adeno-associated viral vectors (which can handle a payload of around 4.7 kb).

The dCas proteins themselves have only minor effects on the regulation of transcription in eukaryotic cells [43]. Due to more complex mechanisms regulating chromatin transcription, significant effects of activation and repression were achieved by fusion of dCas proteins with epigenetic/transcriptional effectors. These effectors can be roughly classified into catalytic and non-catalytic ones (Figure 1B). The former (such as KRAB domains, VP64, etc.) physically interact with endogenous epigenetic machinery to promote transcription activation or repression, while the latter confer epigenetic modification (such as DNA methylation, histone acetylation/deacetylation, etc.) via direct catalytic action on the DNA or histones. The first generation dCas-based systems had a basic architecture (Figure 1C) utilizing simple non-catalytic domains fused to dCas proteins, such as the KRAB domain for repression or VP64 for activation. Such systems allowed for the achievement of 10–100-fold changes in gene expression given constant expression of the dCas systems in the cells [44]. Strategies to develop more potent dCas-based epigenetic regulators are usually based on attaching several epigenetic modulators to the dCas system, often combining different types of domains, including catalytic epigenetic effectors that may confer histone PTMs or DNA methylation. Such examples include the dCas-VPR system where dCas protein fused with VP64, p65 and Rta activation domains [45]. Attaching a greater number of epigenetic effectors also may be achieved through fusing effectors simultaneously to C- and N-termini of dCas (e.g., CRISPRoff, dCas9-2VP) [46,47]. Combination of different epigenetic effectors allows, in some cases, the making of stable epigenetic changes with transient expression by such instruments (e.g., CRISPRoff, KRAB-MeCP2, CHARM) [46,48,49]. Further complexity and potency may be achieved by using advanced architectures based on protein–protein (Figure 1D) and protein–RNA (Figure 1E) interactions. Such systems as CRISPR-SAM or SunTag involve modification of sgRNA by introducing an aptamer sequence or modification of dCas protein by adding a repeating array of peptides that recruit additional activator domains [50,51]. This allows for the maintenance of a reasonable size of each protein/RNA construct while allowing for large complexes to be formed. Such constructs may achieve higher activation or repression levels. The fine tuning and spatio-temporal control of epigenetic engineering may be further achieved by using molecular constructs that interact with endogenous or exogenous effector domains through chemical molecules that may be artificially introduced or removed (Figure 1F) (see a recent review for details [52]).

Without being comprehensive, below we highlight a few of the recent advances and new concepts in the development of dCas-based architectures. Thus, it is important to note the direction devoted to minimizing the size of epigenetic editors and simplifying their delivery into the body. Minimization of the size of epigenetic editors is achieved both by reducing the size of dCas proteins (dCas-MINI, CjCas9) [42,53] and epigenetic domains that strongly increase the size of constructs (for example, the size of the widely used activator domain of VPR is about 1.5 kb). A valid strategy to reduce the size of effector domains is their truncation and identification of sequences directly involved in activation/repression. Thus, a reduced version of VPR (called VPR-mini) was created, which retains its activator activity in different cell types. Another approach is to search for new hypercompact effector domains [54]. The use of high-throughput screening allowed for the identification of activators from the viral interferon regulatory factor 2 (vIRF2) KSHV protein, having sizes up to 96 aa, and longer-term effects comparable in strength to VPR [55].

Combinatorial approaches aimed at signal amplification are being currently developed; for example, the SSSavi platform allows for the recruitment of up to four different effectors [56]. Another example is the improvement of a dCas12a-based activator by creating a split system consisting of two protein fragments, amino (N) and carboxyl (C) fragments of the protein, each carrying two p65-HSF1 domains. The spontaneous dimerization of the N- and C-fragments results in an editor with four copies of the activator domains [57,58]. Interestingly, the expression of a full-length copy of this activator has a much lower activation efficiency. Thus, a similar strategy could be explored for improving other tools based on dCas proteins. Split constructs are smaller in size, which may be beneficial for their delivery. Other works have focused on developing editors based on dCas proteins that utilize novel mechanisms to alter the epigenetic context by fusing them to different domains, both those directly carrying a catalytic function and those auxiliary to it. In one study, for example, the PAD domain from *Porphyromonas gingivalis* (PPAD), which catalyzes the deamination of arginine C-terminal residues, was fused to the N-terminus of dCas9, making possible locus-specific histone citrullination leading to gene activation or repression [59]. In another study a fusion of dCas9 with Matrix metalloproteinase 9 (MMP-9), which catalyzes the proteolytic cleavage of the N-terminal tail of histone H3 (H3NT), was shown to be a novel transcriptional activator [60]. The recently developed epigenetic editing strategy named CHARM utilizes a novel epigenome editing approach that involves the recruitment of endogenous methyltransferases by attaching a DNA-binding protein to the D3L methyltransferase domain which recruits cellular DNMT3A [49]. The tail of histone H3 is also fused to the N-terminus of the editor in the CHARM system. The H3 tail free of the H3K4me3 modification (which is a mark of active chromatin) is needed for DNMT3A activation, which otherwise would remain in an autoinhibitory state. A new epigenetic editing platform called Chem-CRISPR also aims to minimize the expression of exogenous transcription factors within the effector construct and acts as an inhibitor of endogenous epigenetic activators [61]. Approaches have been developed that involve the use of domains that are not in themselves transcription factors but have the potential for multivalent interactions, facilitating the recruitment of additional transcription factors. A number of such domains representing intrinsically disordered regions (IDRs) and modular domains (MDs), as well as combinations of these, have been fused to the widely used VP64 activator, resulting in a significantly increased potency of transcription activation [62]. The modulation of gene expression by affecting G-quadruplexes in promoter regions is also under development. It has been shown that dCas9-based effectors (e.g., fusions with nucleolin) can both increase and decrease gene expression by stabilizing or disrupting the formation of quadruplexes [63,64]. Unexpectedly, the number of studies on the development of tools based on sgRNA modification has been declining recently, possibly due to difficulties in their in vivo application and incomplete understanding of the mechanisms of such regulation.

Epigenetic editors acting by inhibiting proteins with catalytic activity can be singled out as a separate direction. It was shown that recruitment and inhibition of DNMT3A methyltransferase by adding an aptamer to the gRNA sequence leads to gene activation [65]. In another example, the use of computer-aided design enabled the creation of a polycomb repressive complex 2 (PRC2) inhibitor, which also resulted in increased gene activity by removing repressive activity [66].

Taken together, several key directions in the development of new molecular tools for epigenome engineering based on CRISPR-dCas systems can be identified: (1) optimization of the size of the molecular constructs through using smaller dCas proteins and smaller effector domains, so that they would be amenable to relatively simple delivery methods, such as AAV vectors; (2) development of tools that recruit endogenous effector domains, since expression of exogenous effector domains often leads to cytotoxic effects [49,67]; (3) engagement of new epigenetic mechanisms and modifications (e.g., new types of histone PTMs, manipulation of chromatin looping, recruitment of non-coding RNAs and chromatin remodelers, etc.); (4) tuning of dCas-based tools’ efficacy and activity combined with approaches for spatio-temporal control of their action; (5) development of epigenomic editors that act not only by directly changing the state of chromatin, but also through local inhibition of repressor protein functions, which leads to the removal of repression in the target genomic locus [65,66].

## 3. Perspectives of the Applications of dCas-Based Epigenomic Editors in Biomedicine

As previously stated, the epigenome is altered in the vast majority of pathological conditions. It is therefore tempting to consider the possibility of “correcting” the pathogenic changes in the epigenome in humans using epigenomic editors. However, in the majority of diseases, alterations occur not only at the epigenomic level but also affect protein homeostasis, lipid and other low-molecular-weight compound metabolism, intracellular signaling pathways, and complex intercellular interactions. Therefore, modifying the epigenetic state of the genome in a specific region of the genome is not a universal solution. This leads us to view the future of epigenetic editing with a more cautious optimism. Nevertheless, that future is possible. In many cases dCas-based epigenome editing tools have already been applied in proof-of-concept animal disease model studies and at least two clinical trials in humans have been reported (for Duchenne muscular dystrophy and chronic hepatitis B, see below).

In this section, we will examine the potential applications of dCas-based epigenomic editors in various areas of biomedicine by reviewing the recent literature, mainly reporting in vivo studies in animal or human clinical trials (the names of subsections specify the scope). We divided this section into subsections according to the types of diseases or therapeutic modalities, paying attention to describing the nature of the diseases treated by the epigenome editors, so that the mechanism of action and the beneficence of their application becomes evident. In a few cases we also discuss in vitro studies and application of non-dCas-based systems where this is justified for highlighting the future perspectives of dCas-based systems. The list of the discussed studies is given in Supplementary Table S2, and is also available online as an interactive table at https://intbio.github.io/Kovalev_et_al_2025/ST2 (accessed on 24 June 2025).

### 3.1. Hereditary Disorders: Haploinsufficiency (In Vivo Studies)

Haploinsufficiency is a mechanism for the development of genetic diseases when one normal allele is not enough for the gene to function properly. That is, a sufficient dose of the gene is needed for something, and both alleles must be normal. This means that such diseases are inherited in an autosomal dominant manner, and the mutations leading to their manifestation can be either (1) in the regulatory regions of the gene (such as promoter, enhancer, intron, and other regions), leading to decreased gene expression, (2) in the coding region, leading to the production of a nonfunctional protein, and they can also be (3) larger chromosomal abnormalities and deletions [68]. In such diseases, CRISPRa-based editing may be useful to enhance expression of the healthy allele and thus prevent disease manifestation.

Dravet syndrome is a severe epileptic disorder characterized by seizures, and mental and physical retardation, and it usually manifests in the first year of life. It is typically caused by heterozygous mutations in the *SCN1A* gene encoding the alpha-subunit of the Nav1.1 sodium channel, resulting in its haploinsufficiency and disruption of normal neuronal excitability. In one study, newborn mice that carried a mutation in one copy of the *Scn1a* gene were administered the activating construct dCas9-VP160. This has been demonstrated to be an effective method of preventing the development of severe disease manifestations [69]. Another study used transgenic mice that, in addition to a mutation in the *Scn1a* gene, carried dCas9-VPR in their genome, which, due to the Vgat-Cre system, was expressed only in GABAergic inhibitory neurons. These mice were injected with sgRNA-carrying AAVs at 4 weeks of age. The authors were only able to achieve a very modest reduction in disease symptoms in vivo, which they attributed to the adult age of the treated animals [70].

Transcallosal discordance (TD) represents a symptom complex associated with a diverse array of neurodevelopmental disorders. TD is defined by impaired connectivity between the left and right hemispheres of the brain through the corpus callosum, which is caused by hypoplasia resulting from impaired axon growth. One of the genes that may be mutated in a manner that results in this condition is *C11orf46*, which encodes the ARL14EP protein, which is a part of the H3K9 methylation repressor complex. One of the downstream targets of this complex is the *Sema6a* gene, which encodes one of the semaphorins, proteins that control axon growth. Haploinsufficiency of *C11orf46* results in overexpression of *Sema6a*, which can lead to TD. In the study, mice on day 15 of embryonic development were injected with the plasmid via in utero electroporation, which entailed the direct injection of the plasmid into the somatosensory cortex of developing embryos within the uterus of the mother mouse. The plasmid was combined with an activating dCas9-SunTag construct. This editor increased *C11orf46* expression, which in turn lowered *Sema6a* expression, thereby preventing the onset of TD symptoms in newborns [71].

It has been established that haploinsufficiency can result in a number of different forms of hereditary obesity. For example, a heterozygous loss of function of the *SIM1* or *MC4R* gene can result in increased body mass from early childhood. The *SIM1* gene encodes a transcription factor that regulates the development of neurons in the supraoptic nuclei and paraventricular nuclei (PVN) of the hypothalamus. The PVN is a center of satiety in the brain and plays a role in appetite regulation. The *MC4R* gene encodes a melanocortin receptor that transmits satiety signals following food intake [72]. Mathar et al. normalized the phenotype of *Sim1*^+/−^ and *Mc4r*^+/−^ mice by activating a healthy copy of the corresponding gene with dCas9-VP64 in PVN neurons [73]. Therefore, the activation of *SIM1* or *MC4R* expression may represent a potential cure for inherited forms of obesity.

**Figure 2:**
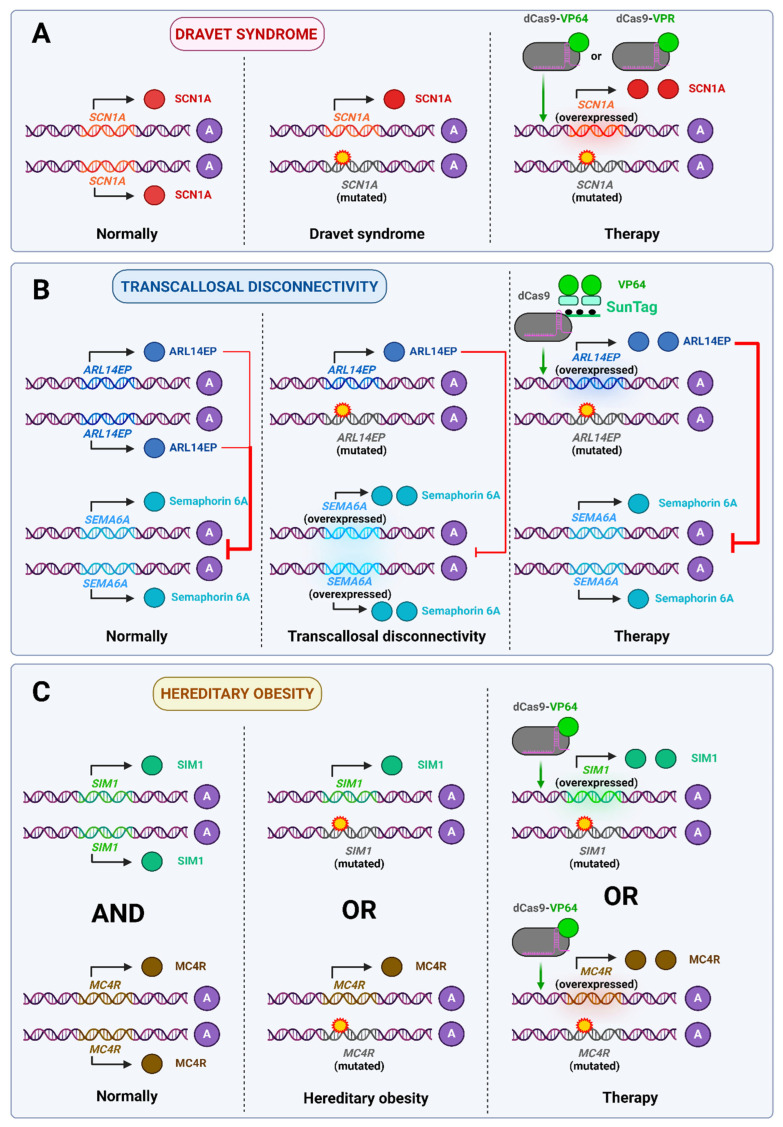
Epigenomic editors can be used to treat haploinsufficiency-related disorders by activating the healthy copy of the gene. (**A**) activation of *SCN1A* can mitigate Dravet syndrome manifestation. (**B**) activation of *C11orf46* can treat transcallosal disconnectivity via *SEMA6A* inhibition. (**C**) activation of either *SIM1* or *MC4R* (dependent of which gene is mutated in the heterozygous way) can rescue hereditary obesity animals’ phenotype. Created with BioRender.com.

These findings demonstrate the efficacy of epigenomic editors in treating diseases caused by haploinsufficiency. However, since normal development (especially of the nervous system) often requires adequate gene expression at a certain period of life, the effectiveness of such therapy may be very limited if the disease is detected late. This is evidenced by two works on Dravet syndrome. These findings highlight the necessity for prenatal diagnosis and extensive genetic screening of embryos. The use of epigenomic editors to treat haploinsufficiency-based disorders is illustrated in Figure 2.

### 3.2. Hereditary Disorders: Imprinting-Related (In Vivo Studies)

Genomic imprinting occurs when only one copy of a gene, obtained strictly from one of the parents, is expressed and the other is silenced. A situation where the paternal copy of a gene is repressed and the maternal copy is active and expressed is called paternal imprinting, the reverse situation is called maternal imprinting. If the copy of the gene that is supposed to be expressed is damaged, it leads to hereditary diseases related to imprinting. Examples include Angelman, Prader–Willi, and Silver–Russell syndromes, and transient neonatal diabetes mellitus. The use of epigenomic editors to treat imprinting diseases by activating a healthy copy of the gene has already been proposed in the literature review [74].

In Angelman syndrome, the maternal copy of the gene-encoding ubiquitin ligase E3A (*UBE3A*) is lost, and the paternal copy of the gene is imprinted and thus not expressed in brain neurons. Loss of *UBE3A* leads to abnormalities in brain development and function, and severe cognitive and motor defects are observed. O’Geen et al. applied ZFP-KRAB to indirectly activate *UBE3A* expression by repressing *Ube3a-ATS*, a long non-coding antisense transcript that represses the paternal copy of the gene. This resulted in restoration of UBE3A protein levels to 25% of normal, as well as recovery of motor abilities to almost the level of healthy mice [75]. Later, Liu et al. used SunTag, which engages DNMT3A and DNMT3L to the region which controls *Ube3a-ATS* expression for the purpose of DNA methylation and gene repression. The therapy alleviated the obesity phenotype in laboratory mice and also improved their motor function compared to the control group [76]. Finally, Li et al. utilized a highly precise CRISPR-Cas13 system that degraded the lncRNA *Ube3a-ATS*, which in vitro resulted in equal expression of paternal and maternal *UBE3A* alleles, and in vivo effects similar to those observed in the previous studies [77].

Loss of other genes at the same locus on the paternal chromosome causes Prader–Willi syndrome (PWS). It is usually associated with a 5.4 or 6 Mb deletion which affects genes such as *SNRPN*, which encodes a protein involved in mRNA splicing, *SNORD116*, which is a cluster of small nuclear RNAs, and others. These are expressed only by the paternal allele, and their loss leads to hypotonia, endocrine disruption, obesity, mental retardation, and other manifestations of PWS [74]. Recently, Rohm et al. made the first attempt to use epigenetic editors to treat this disease. They targeted VP64-dCas9-VP64 and TET1CD-dCas9 to a maternal copy of the *SNRPN* gene (that is healthy and repressed in PWS) in iPSCs derived from patients with PWS. The results were relatively modest, with the authors achieving 5–10% and 10–30% expression of *SNRPN* and its downstream component *SNORD116*, respectively [78]. Although the study was conducted in vitro and exhibited only limited therapeutic effects, it represents a promising initial step toward developing a cure for PWS. Therefore, continued efforts in this direction are warranted.

Imprinting disorders also include, for example, Silver–Russell syndrome. The mechanism is more complex, with an association identified between the condition and abnormality of DNA methylation of the so-called differentially methylated region (DMR). Normally, this region is methylated exclusively on the paternal allele. Loss of methylation leads to increased *H19* gene expression and decreased *IGF2* gene expression. Horii et al. created a mouse model of Silver–Russell syndrome by total demethylation of the DMR [79]. Unfortunately, it is probably impossible to treat this disease by doing the opposite thing, that is, by simply methylating the paternal allele, because of difficulties in distinguishing it from the maternal one. In order to address this challenge, it is necessary to develop new approaches.

**Figure 3:**
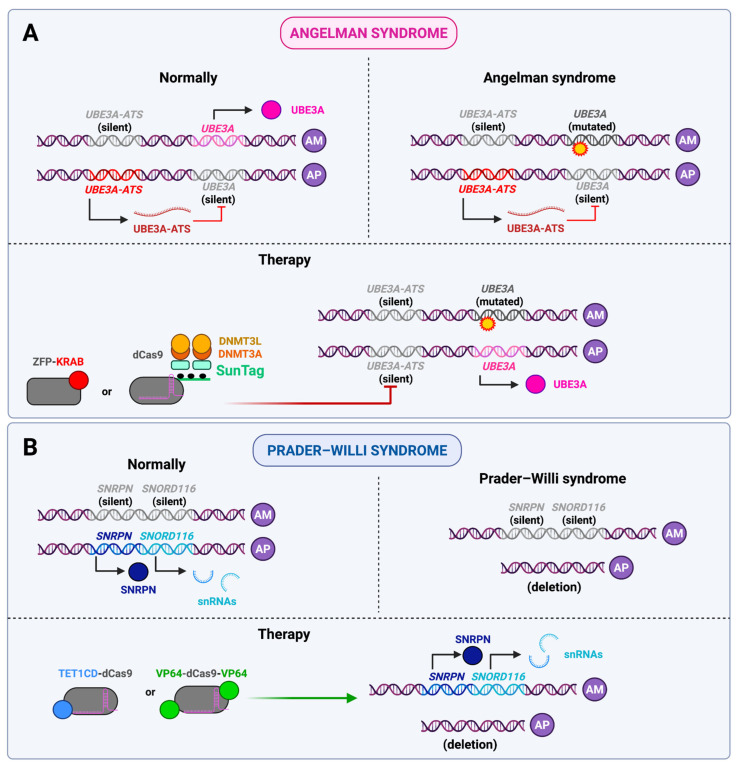
Epigenomic editors can be applied to combat imprinting-related conditions via activation of a healthy, but imprinted, gene copy. (**A**) in Angelman syndrome, activation of paternal copy of *UBE3A* (via *UBE3A-ATS* repression) may be useful. (**B**) maternal *SNRPN*, *SNORD116*, etc. genes could be activated to cure Prader–Willi syndrome. Created with BioRender.com.

Figure 3 illustrates the application of epigenomic editors for addressing Angelman syndrome as a typical imprinting disorder.

### 3.3. Hereditary Disorders: X-Chromosome-Linked (In Vivo Studies and Clinical Trials)

Furthermore, there are a number of X-linked inherited diseases that can be treated with dCas-based therapies. Dystrophies include inherited and acquired diseases associated with underdevelopment or loss of muscle mass. One of the well-known inherited diseases in this group is Duchenne muscular dystrophy (DMD), an inherited progressive disease associated with loss of the gene encoding dystrophin (*DMD*) lying on the X chromosome. Liao et al. activated the expression of the gene encoding utrophin (*UTRN* gene) in dystrophin-deficient mice. Dystrophin is a structural protein that maintains muscle cell integrity during contraction, and utrophin is its close homolog with ~80% similarity. Therefore, *mdx* mice carrying mutation in the *DMD* gene, in which *UTRN* expression was activated, showed partial remission—they had increased muscle mass and grip strength compared to control. It is noteworthy that the activation of the *Klotho* and *follistatin* genes, which regulate cellular metabolism, has also been observed to alleviate the symptoms of DMD in this study [80]. This work had a logical continuation. To cope with delivery issues, Wu et al. created a miniature system, namely MyoAAV-UA, which can be placed in one AAV vector and consists of dCasMINI-VPR (based on dCas12f, which is significantly shorter than the widely used SpdCas9), put under the SPc5-12 promoter (which is known for its heart and skeletal muscle specificity) and targeted at the promoter of the *UTRN* gene. This system was effective across different species: it activated utrophin in cell lines derived from mice, macaques and humans. In iPSC-derived myotubes from DMD patients, the system increased utrophin expression ~3.5-fold, which led to restoration of the utrophin-glycoprotein complex (UGC), which is critical for muscle cell membrane stabilization. In vivo testing was done in mice and *Macaca fascicularis*. In mice utrophin levels increased in skeletal muscles leading to increased muscle strength, while degeneration and fibrosis slowed down. The effects were stable for 6 months. In *Macaca fascicularis* the effects were similar, and it happened without immune reaction and only a slight and transient increase in the levels of ALT/AST was observed [81]. Thus, in the presence of certain mutations, activation of a homologous gene may prove to be an effective treatment strategy for X-chromosome-linked diseases in male patients.

Martin–Bell syndrome, also referred to as fragile X syndrome (FXS), is a sex-linked disorder and represents the most prevalent form of mental retardation in males. This is due to the fact that it is a sex-linked disorder. The condition is caused by an expansion of CGG repeats in the promoter region of the *FMR1* gene. In individuals without the condition, the number of repeats ranges from 6 to 44. However, in patients with FXS there are more than 200 repeats. This increase in the number of repeats results in promoter hypermethylation and a reduction in *FMR1* gene expression. Given that the product of this gene plays a role in regulating the development of neurons and synaptic plasticity, these changes contribute to abnormalities in the formation of nervous system architecture. In the study, fibroblasts were isolated from FXS patients, from which induced pluripotent stem cells (iPSCs) and, subsequently, neurons were obtained. The demethylation of the *FMR1* promoter with dCas9-TET1 at the stage of both iPSCs and neurons resulted in an increase in the expression of this gene and the restoration of the normal phenotype of neurons. This was maintained when the neurons were transplanted into the mouse brain, indicating that the phenotype was stable in vivo [82]. Interestingly, it was not possible to obtain a mouse model of FXS: the presence of even more than 300 repeats in the promoter of the *FMR1* gene did not lead to its hypermethylation, and such animals did not have a phenotype similar to FXS patients [83]. Therefore, in order to transfer the in vitro model to clinical practice, another animal in vivo model, different from the mouse one, must be created, or an attempt should be made directly on patients.

Rett syndrome is associated with the loss of function of the methyl CpG-binding protein 2 (*MECP2*) gene and predominantly affects girls. In the heterozygous state, it causes loss of speech and motor skills after 6–18 months of age, as well as severe mental retardation and stereotyped hand movements. This is due to the mosaicism of *MECP2* expression: it occurs in about half of the cells in the body. In males, the presence of a single X chromosome results in a more pronounced manifestation of the mutation, leading to a range of severe symptoms, including neonatal encephalopathy, ataxia, tremor, and others [84]. Qian et al. created a potential therapy for Rett syndrome and demonstrated its efficacy in vitro. First, demethylation of the *MECP2* promoter with dCas9-TET1 in human embryonic stem cells allowed for the obtention of neurons with soma of normal size and without electrophysiological abnormalities, which means normalization of the phenotype. Furthermore, the combined use of dCas9-TET1 to activate *MECP2* and of dCpf1-CTCF to isolate the *MECP2* locus (in order to prevent the spreading of inactivating chromatin tags) on either side of *MECP2* resulted in an increase in its expression from 30% to 59% of normal values and a further alleviation of phenotypic morbidities [85].

The *CDKL5* gene encodes cyclin-dependent kinase-like 5, the loss of function of which leads to epilepsy beginning in the first months or years of life. As in the case of Rett syndrome, this disease in the heterozygous state affects girls more often, while boys are afflicted less commonly but more severely [86]. Halmai et al. applied dCas9-TET1 and dCas9-VP64 to activate *CDKL5* expression in the SH-SY5Y neuroblastoma cell line, which resulted in phenotype normalization. Moreover, the simultaneous use of both editors gave a more significant result than using them separately, and a synergistic effect was noted [87].

Accordingly, the extant research in this domain can be classified into two categories. In the first case, as in FXS, the disease is associated with reduced gene expression (but the resulting protein product is normal) and affects boys. The solution, therefore, is to artificially bring the expression to normal. In the second case, when the mutation inactivates the protein and affects girls in a heterozygous state, as in Rett syndrome and infantile epilepsy, the objective is to activate the expression of the healthy allele in all cells of the body. This can be considered to be analogous to the treatment of haploinsufficiency diseases. It is noteworthy that a strategy employing a CTCF-based editor to isolate the edited region from the surrounding chromatin has been developed, which is particularly relevant in the context of the inactivation of a single X chromosome in females. It remains to be seen what the appropriate course of action should be if the protein product of the sole gene in boys is disrupted.

**Figure 4:**
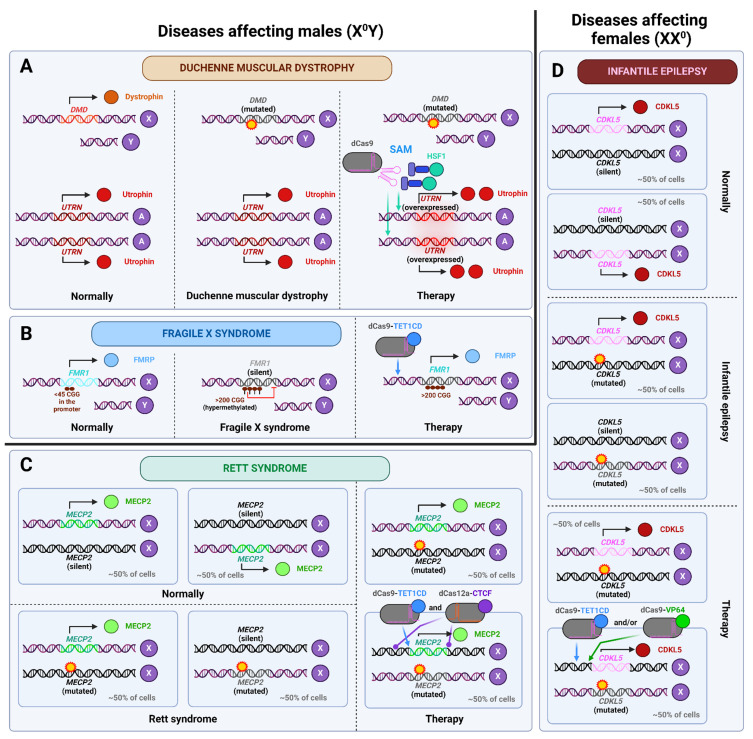
Possible applications of epigenomic editors to treat X-chromosome-linked disorders. (**A**) in Duchenne muscular dystrophy, the only copy of *DMD* in males is lost, so activation of its analogue *UTRN* may be helpful. (**B**) in Fragile X syndrome, promoter demethylation. and thereby activation of *FMR1*, might rescue the disease phenotype. (**C**) in Rett syndrome in females, activation of the healthy copy of *MECP2* can be considered as a curative method. (**D**) activation of *CDKL5* in infantile epilepsy girls can mitigate the disease manifestations. Created with BioRender.com.

The application of epigenomic editors in treating X-chromosome-linked disorders is depicted in Figure 4.

### 3.4. Hereditary Disorders: Recessive Autosomal (In Vivo Studies)

Epigenomic editors may also be employed to treat heritable disorders with a recessive autosomal mode of inheritance, provided that the mutation in question affects the gene expression level and not the protein product itself, or provided that it is feasible to activate a homologous gene with a comparable function.

One illustrative example of the first strategy is the epigenetic treatment of Friedreich’s ataxia, a disease of the nervous system and heart that typically manifests in childhood or adolescence and is characterized by disorders of coordination, balance, and speech, as well as cardiomyopathies. Friedreich’s ataxia is caused by a deficiency in the protein frataxin, which plays a key role in mitochondrial iron metabolism, so its deficiency leads to mitochondrial dysfunction, oxidative stress, and other issues. In 96% of patients, the disease develops as a consequence of the expansion of the GAA repeats from their normal values of 5–33 to 66–1300 in both alleles of the first intron of the *FXN* gene. This results in a reduction in the expression of this gene [88,89]. TALE-VP64 targeting 14-bp DNA sequence in the promoter of *FXN* was created, and it increased *FXN* mRNA levels by 2- to 3-fold in 293FT cells [90] and by 2-fold in human fibroblasts derived from human Friedreich’s ataxia patients [91]. The same artificial transcription factor (ATF) was then applied in vivo on YG8R mice aged 9 days and 4 months. AAV9 encoding this genetic construction was injected intraperitoneally, which led to increased *FXN* expression in heart, skeletal muscle and liver. However, no behavioral or other tests have been performed to determine whether this therapy helps to avoid the phenotypic manifestation of the disease [92]. A completely different approach involves the use of sequence-specific synthetic transcription elongation factors (Syn-TEFs), which allow the transcription across repressive chromatin to go. Syn-TEF1 was developed with the objective of enabling RNA polymerase II to traverse GAA repeats in the *FXN* gene. This objective was achieved in vitro in multiple patient-derived cell lines. As can be observed, the mechanism of action of Syn-TEFs is fundamentally distinct from that of any ATFs. Syn-TEFs comprise a DNA-binding component and a protein that activates the transcription mechanism, so we will not discuss it in more detail [93]. To summarize, there are already several approaches to the treatment of Friedreich’s ataxia, both TALE-VP64-based and Syn-TEF-based.

As previously discussed, one potential avenue for treating DMD is to activate utrophin, a homolog of the damaged dystrophin gene. A further example of this approach is the treatment of congenital muscular dystrophy type 1A (MDC1A), an autosomal recessive disorder resulting from mutations in both copies of the *LAMA2* gene. The protein encoded by this gene, laminin-α2, forms a heterotrimer with the β1 and γ1 chains to create the laminin-211 complex, which is crucial for the interaction between muscle cells and the extracellular matrix. Deficiency of laminin-α2 results in the disruption of interactions between muscle fibers and integrins and α-dystroglycan, as well as impaired differentiation of Schwann cells, amongst other effects. The extensive range of *LAMA2* mutations precludes the possibility of a single gene therapy for MDC1A. Consequently, Kemaladewi et al. employed a strategy analogous to that previously discussed in the context of DMD, whereby the expression of *Lama1* (a homolog of *Lama2*) was activated in mice. The VP64-dSaCas9-VP64 construct was used for this purpose, with the sgRNA under its promoter packaged into a single AAV9 vector for delivery. This therapeutic approach, even when initiated after the onset of symptoms, has been shown to prevent the development of muscle fibrosis and paralysis. Consequently, it may be regarded as a promising avenue of treatment for MDC1A [94].

One of the oldest attempts to use epigenomic editors was to treat β-thalassemia and sickle cell disease (SCD). Both of these diseases are caused by mutations in the β-globin gene (*HBB*), which encodes two of the four hemoglobin chains, with beta-thalassemia being caused by various loss-of-function mutations that result in decreased expression or loss of function of the protein, and SCD being caused by a point mutation that results in the substitution of glutamate for valine in the active center, which affects the structure of hemoglobin and ultimately changes the shape of red blood cells. Both β-thalassemia and sickle cell disease (SCD) are autosomal recessive diseases, although heterozygous carriers may have milder symptoms. And both of these diseases can be treated by activating a homologue of β-globin that is not normally expressed in adult humans. ZFP-VP64 increases fetal γ-globin (*HBG1*) expression ~10–16-fold in vitro in K562 hematopoietic cells and healthy CD34+ cells [95,96]. Although fetal hemoglobin activation may improve oxygen transport in patients with these diseases, it has not progressed beyond these studies. Instead, a therapy using CTX001 (Casgevy™, manufactured by Lonza, Geleen, The Netherlands), which relies on ex vivo autologous CD34+ hematopoietic stem- and progenitor cell-editing with CRISPR-Cas9, has been proposed [97]. Moreover, clinical trials of this drug against β-thalassemia (NCT03655678) and SCD (NCT03745287) are already underway, and it is the only FDA-approved cell therapy that applies CRISPR-based genome editing [31].

**Figure 5:**
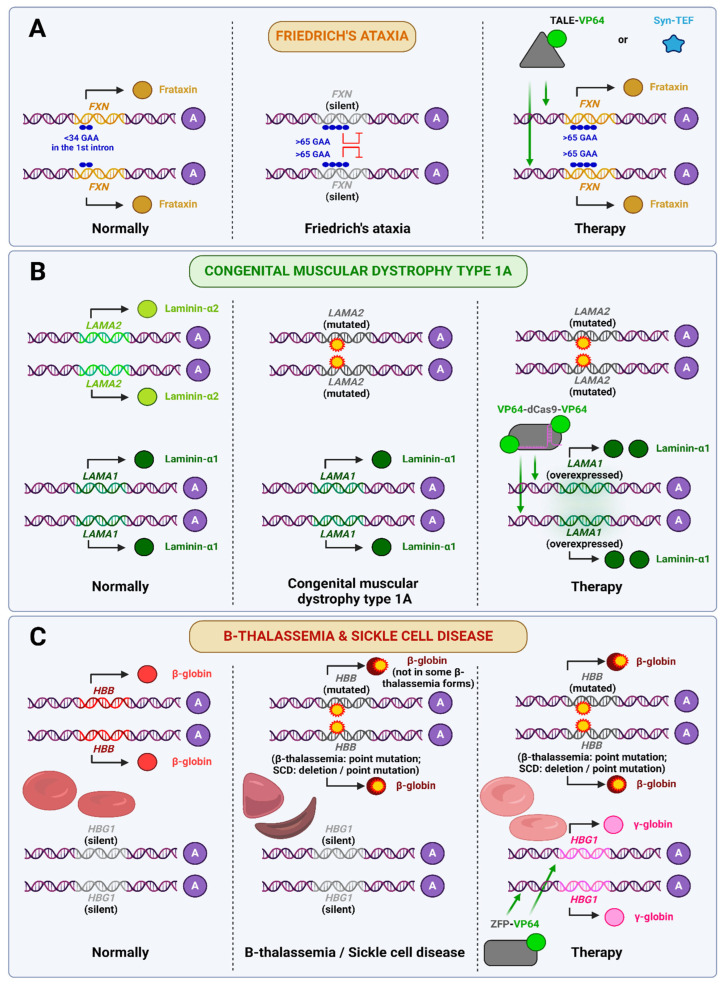
Epigenomic editors can be a future medication for some autosomal recessive conditions. (**A**) activation of *FXN* expression can reverse its downregulation in Friedrich’s ataxia and remove its symptoms. (**B**) in MDC1A, *LAMA2* is lost, so activation of its homologue *LAMA1* can improve the condition of such patients. (**C**) activation of *HBG1* can be used as a treatment for β-thalassemia and sickle cell disease where healthy *HBB* is absent. Created with BioRender.com.

As shown in Figure 5, epigenomic editors are utilized to manage disorders stemming from autosomal recessive mutations.

### 3.5. Neurodegenerative Diseases (In Vitro and In Vivo Studies)

In general, neurodegenerative diseases are slowly progressing disorders that are characterized by the gradual death of neurons, which ultimately leads to cognitive and motor impairment. In many cases, such as in Parkinson’s and Alzheimer’s diseases, these changes are accompanied by an abnormal accumulation of a particular protein, which forms aggregates. Given this, it seems reasonable to posit that CRISPRi could be used to repress the corresponding gene and halt the progression of the disease. In this section, we will examine how this idea is realized in the available studies.

Huntington’s disease, or Huntington’s chorea, is a fatal neurodegenerative disease inherited dominantly and associated with expansion of CAG repeats in the *HTT* gene, which encodes the protein huntingtin. Normally there are 10 to 29 such repeats, but if there are 36 or more, the disease manifests in middle age. Mutant *HTT*, containing elongated polyglutamine (polyQ) regions, precipitates and causes neuronal death, whereas normally this protein is required for the maintenance of neuronal processes. In two studies, ZFP-based epigenomic editors were used to suppress the expression of the mutant *HTT* allele in heterozygous mouse models. In their first paper published back in 2012, the authors created several editors containing different amounts of zinc fingers as well as a KRAB domain for gene repression. They were able to achieve repression of mutant *HTT* up to 78% in vitro in the STHdh cell line and up to 60% in vivo in the R6/2 mice (which harbor 115–160 CAG repeats). This resulted in serious symptom mitigation, with no significant reduction in expression of the normal *HTT* allele, striatum volume, or cell density in the striatum, indicating the safety of this therapy [98]. In a later study, 41 candidate editors based on ZFPs (also carrying the KRAB domain) were tested, which allowed for selection of the most selective ones, achieving suppression of nearly 100% of the mutant *HTT* while maintaining almost 90% expression of the normal allele. The results demonstrated the absence of significant toxicity and neuroinflammation following the administration of the editor for a period of nine months. Furthermore, the work was supported by commercial companies, such as the CHDI Foundation and Shire Human Genetic Therapies, Inc., which lends credence to the possibility of implementing this approach in clinical practice in the near future [99].

Among the most lethal, albeit uncommon, neurodegenerative disorders are prion diseases, including Kuru disease, Creutzfeldt–Jakob disease, fatal familial insomnia, and others. These disorders are all associated with neuronal death due to the shedding of prion protein aggregates, which can be random, caused by mutations, or the result of ingestion of this protein. Neumann et al. designed and applied the epigenomic editor CHARM (consisting of ZFP/TALE/dCas9, DNMT3L, and histone H3 tail, the latter two elements being required for efficient recruitment of endogenous DNMT3A) to methylate the *Prnp* promoter, resulting in an 80% reduction of prion protein levels in mouse brains [49]. It is regrettable that the mice utilized in the experiment were initially healthy, and the sole outcome was a decline in protein levels. Consequently, it remains unclear whether this therapeutic approach will prove effective in combating a prion disease that is already developing.

Alzheimer’s disease is a neurodegenerative condition that occurs in elderly age and is characterized by progressive loss of memory and cognitive function. In addition to chronic inflammation and microglia activation in the brain, two key proteins are associated with its development: (1) β-amyloid (βA), which is formed from amyloid precursor protein (APP) and then precipitates and forms plaques between neurons, and (2) tau, which normally maintains normal microtubule structure but in Alzheimer’s disease becomes hyperphosphorylated (TauHF) and aggregates within neuronal bodies. Wegmann et al. reduced the expression of the *MAPT* gene encoding the tau protein in the APP/PS1 mouse model of Alzheimer’s disease by using ZFP-KRAB. This reduced tau protein levels and protected the mice’s neurons from damage, and the effect of a single injection was maintained for 11 months. This could be a promising therapy for the treatment of Alzheimer’s disease, although the authors did not perform behavioral tests in mice to assess phenotypic changes [100]. Another interesting approach was applied by Fabio Duarte in his thesis research. He activated the *ADAM9*, *ADAM17* and *TFEB* genes using dCas9-VPR [101]. ADAM9 and ADAM17 are α-secretases and cleave APP such that non-amyloidogenic s-APPα is formed, and this pathway competes with sequential cleavage by β- and γ-secretases leading to βA formation [102]. At the same time, TFEB acts as a key positive regulator for a variety of lysosomal genes; its upregulation enhances the clearance of intracellular pathogenic forms of Aβ and TauHF via autophagy [103]. Probably, this strategy, as well as a decrease in *APP* or *BACE1* (which encodes β-secretase) expression, will be helpful in treating Alzheimer’s disease.

The second most prevalent neurodegenerative disorder after Alzheimer’s disease is Parkinson’s disease. It typically manifests in advanced age and is characterized by a gradual decline in motor function due to the death of dopamine neurons in the substantia nigra of the midbrain. The molecular causes of Parkinsonism encompass the deposition of alpha-synuclein aggregates within neurons, the formation of Lewy bodies, mitochondrial dysfunction and oxidative stress, microglia activation and neuroinflammation, and other factors. Currently, there is no effective therapy for Parkinsonism, and the drug L-DOPA, which is a precursor of dopamine, is used as maintenance therapy [104,105]. An intriguing solution was proposed by Giehrl-Schwab et al., who successfully reprogrammed astrocytes into GABAergic neurons in vivo within the striatum. This was accomplished using the SAM system, which activated *Ascl1*, *Lmx1a*, and *Nr4a2*, transcription factors associated with neurogenic differentiation. This therapy resulted in a significant improvement in motor activity in mice treated with 6-OHDA, a toxin that induces the death of dopamine neurons. New GABAergic neurons were effectively incorporated into the neuronal network of the striatum and effectively compensated for these losses [106]. Another approach was used by Guzmán-Sastoque et al., who conducted an in vitro study on a mixed culture of neurons, astrocytes, and microglia derived from newborn rat brains and treated with MPTP, a neurotoxin that causes Parkinson’s-like symptoms. The authors activated the *GDNF* gene 200-fold in such a culture using CRISPR-SAM. *GDNF* is a neuroprotective gene, and its activation led to a decrease in the ROS level in the cells, a decrease in the activity of MAO-B, an enzyme that destroys dopamine, and, finally, to an increase in cell survival by 34.6% [107]. It would be of interest to explore the possibility of decreasing the expression of *SCNA*, which encodes α-synuclein [108], or increasing the expression of the neuroprotective gene *Nrf2*, whose product reduces ROS levels in neurons and is thus neuroprotective [109], particularly given that *Nrf2* has already been activated in the brains of healthy mice by dCas9-VP64 [110] to combat Parkinson’s disease.

It is well-established that the human eye contains three types of cone cells, which are responsible for perceiving different colors. These cells are known as “blues”, “greens”, and “reds”, and they utilize specific types of opsins to facilitate their function. The “blues” utilize S-opsin, the “greens” synthesize M-opsin, and the “reds” employ L-opsin. In all four cases, the opsin, when activated by a quantum of light, activates the G-protein transducin, which then degrades cGMP. This leads to the closure of the (CNGA)x3CNGB channel and hyperpolarization of the cell, which is a signal [111]. Retinitis pigmentosa, an inherited disorder with an incidence of 1 in 4000 individuals, is characterized by the gradual death of first the rods and then the cones, developed by a somewhat similar mechanism to neurodegenerations [112]. The disorder is heterogeneous and caused by mutations in approximately 200 genes. Among the most common are dominant mutations in the *RHO* gene, which encodes rhodopsin, such as P23H. Dominant mutations in *RHO* lead to improper folding of this protein, which, in turn, causes endoplasmic reticulum stress, impaired transport, retinal inflammation, and photoreceptor death; retinitis pigmentosa can also be caused by *RHO* deficiency or mutations in other genes [113,114]. To prevent photoreceptor death in rhodopsin-deficient mice, Böhm et al. used dCas9-VPR to activate *Opn1mw*, which encodes M-opsin, and *Cnga1*. The resulting mice exhibited a notable reduction in neuronal death and retinal degeneration 12 months after this therapy, thereby indicating that it is both highly efficacious and safe [115]. An alternative approach was employed by Moreno et al., who utilized KRAB-dCas9 to inhibit the expression of *Nrl*, a transcription factor that stimulates rod differentiation and suppresses cone differentiation. This *Nrl* knockdown resulted in the direct reprogramming of rods into cones and the protection of mice from vision loss [112].

The potential of epigenomic editors to treat neurodegenerative diseases is, in our estimation, particularly promising, given the inevitability of these disorders and the limitations of existing therapies. This assertion is corroborated by the results of clinical trials of a drug designed to treat Huntington’s chorea. A significant number of approaches in this field, including those put forth by our own research group, remain at the conceptual stage. This creates a vast scope for future researchers and clinicians to explore and expand upon these concepts.

**Figure 6:**
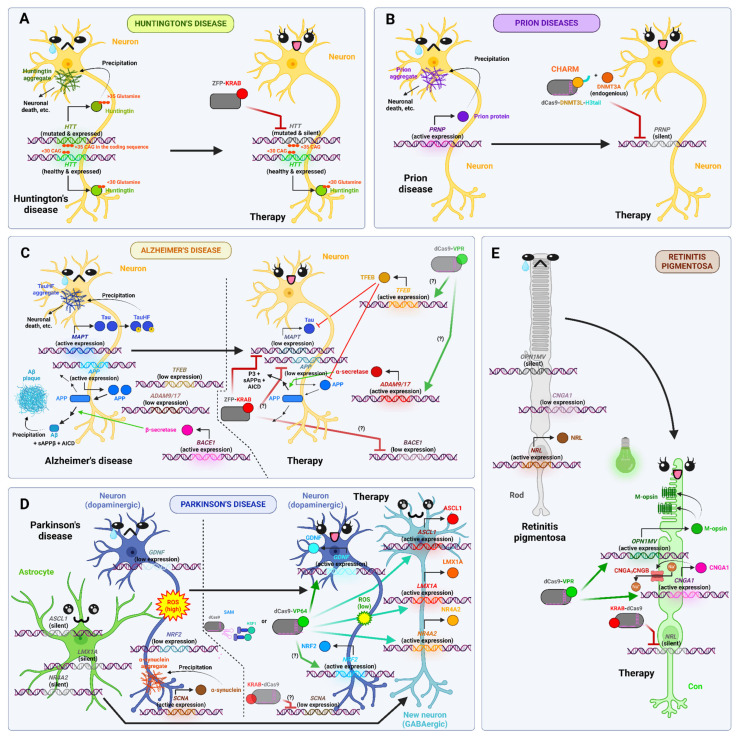
Epigenomic editors can prevent the onset of neurodegenerative disorders. (**A**) repression of mutated copy of *HTT* can prevent huntingtin aggregates formation and save Huntington’s disease patients. (**B**) repression of *PRNP* expression can prevent neuronal death because of prion aggregates. (**C**) to treat Alzheimer’s disease, we can (1) repress *MAPT* to diminish TauHF aggregation; (2) repress *APP* to avoid Aβ plaque formation; (3) decrease *BACE1* and increase *ADAM9/17* expression to direct APP cleavage through a non-amyloidogenic pathway; (4) activate *TFEB* expression to accelerate protein cleavage. (**D**) to treat Parkinson’s disease, we can (1) activate *GDNF*, *NRF2* and other neuroprotective genes to decrease ROS level and neuronal mortality; (2) repress *SCNA* to avoid α-synuclein aggregate generation; (3) activate *ASCL1*, *LMX1A* and *NR4A2* in astrocytes to reprogram them into neurons in vivo. (**E**) to avoid retinal degeneration in retinitis pigmentosa patients, we can: (1) repress *NRL* for direct reprogramming of rods into cones; (2) activate *OPN1MV* and *CNGA1* to better capture light quants. “(?)” indicates some future directions for which no evidence in the context of epigenomic editors was obtained yet. Created with BioRender.com.

Figure 6 demonstrates how epigenomic editors can be used to treat disorders associated with neurodegeneration.

### 3.6. Psychoneurological Conditions and Addictions (In Vivo Studies)

A very promising area of application for epigenomic editors could be neurological, mental illnesses and addictions; for example, for the treatment of excessive alcohol consumption and increased anxiety in adults following adolescent alcohol exposure. At the molecular level, this behavior is caused by a reduction in the expression of the activity-regulated gene, the cytoskeleton-associated protein gene (*Arc*), within the central nucleus of the amygdala. To enhance *Arc* expression in a rat model, dCas9-p300 was directed towards the so-called synaptic activity response element (SARE), which is situated in the *Arc* enhancer. This resulted in an increase in the expression of enhancer RNAs (eRNAs) encoded in the SARE region, which facilitated the removal of negative elongation factor (NELF) from the *Arc* promoter. This, in turn, led to an increase in the latter’s expression and a decrease in anxiety and craving in the animals. In contrast, the repression of *Arc* by dCas9-KRAB resulted in increased anxiety and alcohol craving, thereby further confirming the link between these manifestations and *Arc* expression. In light of the lack of efficacious treatments for alcohol use disorder, this therapeutic approach may offer a promising avenue for further investigation. The sole disadvantage of this approach is the utilization of mutagenic lentiviral vectors due to the considerable size of p300 [80]. Consequently, we propose the consideration of the SadCas9-VP64 editor, which can be packaged into an AAV vector, for the activation of Arc expression.

Another illustrative example is chronic pain, which develops as a consequence of slow-healing wounds, chronic inflammation, cardiovascular disease, diabetes, cancer, and other conditions, and affects between 19 and 50% of the global population. Currently, treatment options include the use of either opioids, which have the potential for addiction, or lidocaine, which non-selectively blocks a significant number of sodium channels and may result in adverse neurological effects. The NaV1.7 channel, which is encoded by the *SCN9A* gene, is expressed in nociceptors. Loss of the *SCN9A* gene results in rare inherited forms of pain insensitivity. Consequently, Moreno et al. devised the ZFP-KRAB and KRAB-dCas9 systems targeting *Scn9A* and employed them to treat three models of chronic pain in mice: (1) carrageenan-induced inflammatory pain, (2) paclitaxel-induced neuropathic pain, and (3) BzATP-induced pain. In all three cases, epigenetic therapy resulted in a significant reduction in pain sensitivity, approaching normal levels. No adverse effects were observed in relation to impaired motor function, and the analgesic effect was maintained for a minimum of 308 days for inflammatory pain and 105 days for polyneuropathic pain. This offers promising prospects for the development of a drug that can provide long-term and reliable pain relief with a single injection [116].

A significant challenge is epilepsy, which presents as unprovoked seizures and affects approximately 1% of the global population. Approximately 30% of patients do not respond to existing pharmacological interventions. Colasante et al. developed a mouse model of epilepsy by injecting kainate, which is an agonist of the corresponding glutamate receptors in the brain. In high doses, kainate causes status epilepticus, an acute condition with symptoms similar to those observed in epilepsy. To treat the animals, they were administered an injection of dCas9-VP160, which activates the *Kcna1* gene, one week later. The gene in question encodes a subunit of the potassium channel Kv1.1, which plays a role in the repolarization of neurons and reduces their excitability. The incidence of epileptic seizures was reduced in the treated mice compared to the control group, and cognitive function was also improved [117].

**Figure 7:**
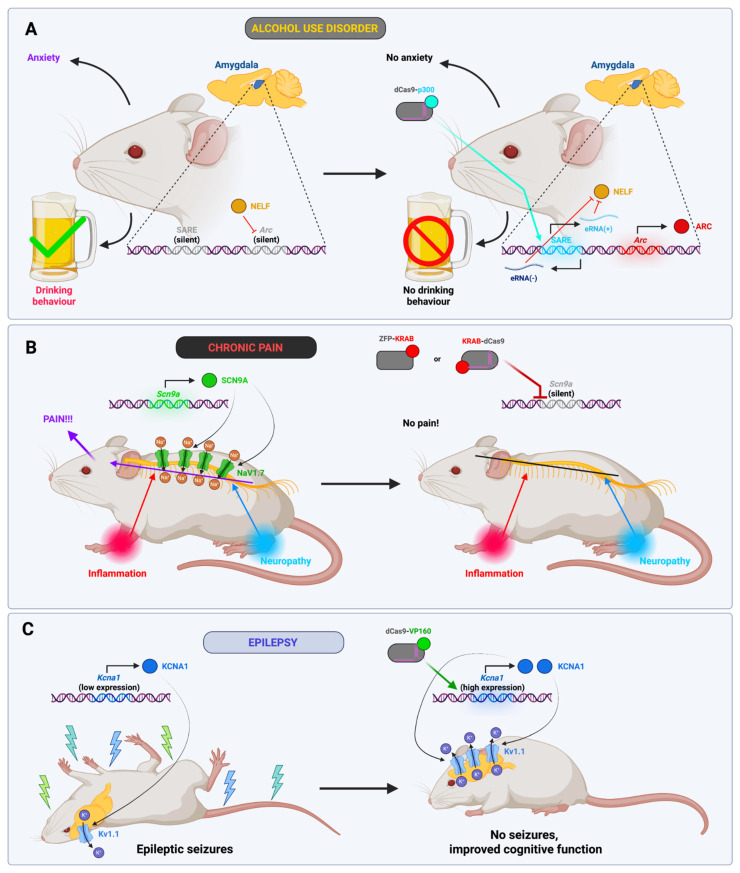
Epigenomic editors can be employed to combat addictive, psychiatric, and neurological pathologies. (**A**) activation via acetylation of SARE and thus activation of *Arc* gene in brain amygdala can remove drinking behavior and anxiety following adolescent alcohol exposure. (**B**) inhibition of *Scn9a* which encodes a subunit of sodium channel can hinder its assembly and thereby chronic pain transmission into the brain. (**C**) activation of *Kcna1* expression which encodes a subunit of potassium channel can reduce neuronal excitability and prevent epileptic seizures. Created with BioRender.com.

The treatment of neurological and psychiatric disorders, and addictions using epigenomic editors is represented in Figure 7.

### 3.7. Metabolic Diseases (In Vivo Studies)

Cardiovascular disease (CVD) represents the leading cause of mortality worldwide, accounting for approximately 32% of all deaths. One of the most prevalent forms of CVD is atherosclerosis [118,119]. Atherosclerosis is a disease of the arterial walls that is caused by the accumulation of cholesterol, which results in the formation of plaque, chronic inflammation, and calcification. This leads to the loss of arterial elasticity, increased fragility, and a reduction in the lumen. Atherosclerosis is a disease that typically manifests in individuals of advanced age. The principal risk factor for the development of atherosclerosis is elevated levels of low-density lipoprotein (LDL) in the blood, which contains a substantial quantity of cholesterol. Low-density lipoprotein (LDL) is removed from the bloodstream by liver cells following its binding to receptors on the surface of hepatocytes. The enzyme protein convertase subtilisin/kexin type 9 (PCSK9) is secreted from liver cells into systemic circulation and is also capable of binding to LDL receptors, thereby promoting their destruction. Accordingly, one potential strategy for reducing LDL levels is to reduce PCSK9 levels, for instance, through the use of monoclonal antibodies [83,84]. The CRISPR/Cas9 system has also been employed for this purpose. *PCSK9* has been targeted by both the Cas9 nuclease, which results in gene knockout [85], and an adenine base editor, which alters the splicing of its mRNA in primates [120]. With regard to the use of epigenomic editors, Thakore et al. employed a dual-vector AAV8 system targeting hepatocytes and expressing dead *Staphylococcus aureus* Cas9 fused to the KRAB domain (SadCas9-KRAB) and *PCSK9*-targeting gRNA. SaCas9 was selected for its smaller size, which avoids issues with AAV packaging. The system demonstrated a capacity to reduce blood PCSK9 levels by up to 10% of the baseline, with some of this reduction remaining stable over a 24-week period. The reduction in LDL levels was less pronounced, at approximately 55%, and the effect was less sustained [87]. The objective of the second study was to optimize this approach. First, the flexible glycine-rich linker and SV40 nuclear localization signal (NLS) were replaced with a rigid helical linker and c-Myc NLS, respectively. This resulted in a significant increase in the SadCas9 KRAB protein’s nuclear entry from 6.7% to 27% in the ARPE-19 cell line. Furthermore, positioning the KRAB domain at the N-terminus, rather than the C-terminus, led to an additional enhancement in nuclear localization. Secondly, the promoters for both the gRNA and the KRAB-dSaCas9 were optimized and minimized, thus allowing the entire construction to be packaged into a single AAV2/8 instead of a dual system [88]. Regarding the disadvantages of such an approach to combat hypercholesterolemia, elevated blood levels of alanine transaminase (ALT) indicated hepatotoxicity, detected in the first study. The reason for this may be an immune reaction against the SaCas9 protein, which was detected by RNA sequencing [121]. A more detailed discussion of the issue of Cas protein immunogenicity and delivery can be found in Section 7.4.

Myocardial hypertrophy can be either physiological (e.g., in athletes) or pathological (e.g., in hypertension or ischemic heart disease). It is a risk factor for heart failure and myocardial infarction. The overexpression of *Mef2d* by dCas9-VPR in cardiomyocytes using the *Myh6* promoter resulted in the development of myocardial hypertrophy in transgenic mice [122]. It is likely that suppression of *MEF2D* may be useful in controlling this disease.

As previously discussed, the treatment of hereditary dystrophies using epigenomic editors has been explored. This discussion will now turn to the treatment of acquired dystrophies. Liao et al. demonstrated that *Fst* activation can reduce the manifestations of DMD and increase muscle mass and strength in healthy mice when injected into the corresponding muscle [80]. The potential use of doping in sports will not be addressed in this discussion. However, this technology may have two significant applications in the treatment of acquired dystrophies. The first area of potential application is the acceleration of muscle recovery following injuries such as fractures and sprains, which result in prolonged periods of disability and muscle degeneration [123]. The second point pertains to the fact that muscular dystrophy is a condition that commonly manifests with advancing age. This is particularly associated with the loss of mobility among the elderly [91]. It is therefore conceivable that this process could be reversed.

Type 1 diabetes mellitus is a chronic disease resulting from the autoimmune destruction of pancreatic insulin-producing cells, typically manifesting during adolescence. At present, insulin therapy represents the most prevalent method for maintaining these patients. Liao et al. employed the SAM system to stimulate the expression of the *Pdx1* gene, a pivotal regulator of beta cell differentiation, in hepatocytes of a mouse model of streptozotocin-induced diabetes. The mice exhibited enhanced capacity to regulate blood glucose levels, accompanied by increased expression of *Ins1* and *Ins2* genes, which encode insulin, and *Pcsk1* in hepatocytes. However, the authors did not conduct histological or other studies to ascertain the presence of fully functional beta cells in the liver tissues [80]. Although such therapy may be theoretically promising, at present, an approach based on the transplantation of induced pluripotent stem cell (iPSC)-derived beta cells (as part of what is known as a stem cell islet) into the patient’s body seems to offer a more promising avenue of investigation [124,125]. A recent case study has demonstrated that a patient with type 1 diabetes has exhibited sustained remission of the disease for a period of one year following the initiation of a therapeutic regimen [126].

**Figure 8:**
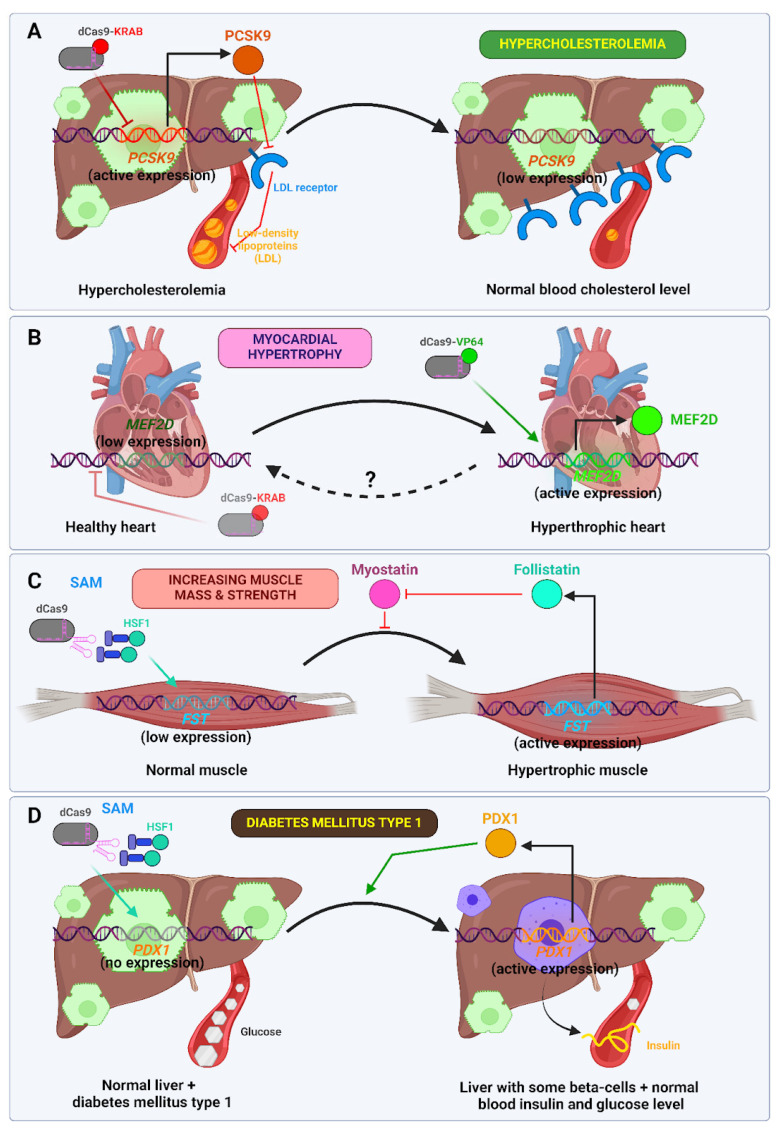
Epigenomic editors can improve metabolic related disorders. (**A**) Repression of the *PCSK9* gene in the liver can decrease LDL (“bad cholesterol”) levels in blood and thus prevent atherosclerosis. (**B**) activation of *MEF2D* in the myocardium can cause myocardial hypertrophy, and it is probable that repression of *MEF2D* may be useful to combat this condition. (**C**) activation of the *FST* gene in muscles can increase their mass in strength, including in healthy animals. (**D**) activation of the *PDX1* gene in some liver cells can make them insulin-producing and rid the patient of type 1 diabetes. “?” indicates a potential opportunity to suppress *MEF2D* expression as a therapeutic approach to counteract hypertrophy (this has not been demonstrated experimentally. Created with BioRender.com.

In Figure 8, the use of epigenomic editors for the treatment of metabolic conditions is summarized.

### 3.8. Autoimmune Disorders (Only In Vitro Studies Yet)

Epigenomic editors have recently been applied to reverse the changes caused by autoimmune disorders, yet these studies are only done ex vivo so far.

Systemic lupus erythematosus (SLE) is a chronic autoimmune disease characterized by aberrant activation of the type I interferon (IFN) pathway and loss of immune tolerance. A critical regulator of this pathway, miR-146a, is downregulated in SLE, contributing to uncontrolled inflammation. While genetic variants near miR-146a have been linked to increased SLE risk, the epigenetic mechanisms driving its suppression remain unclear.

In a 2023 study published in Arthritis & Rheumatology, Zhu et al. combined 3D genomics, epigenomic profiling, and CRISPR-based editing to identify and target a dysfunctional enhancer of miR-146a in immune cells [127]. Using circular chromosome conformation capture (4C-seq) and ATAC-seq, the authors mapped chromatin interactions and accessibility in monocytes, B cells, and T cells from SLE patients and healthy donors. They discovered that an enhancer located 32.5 kb downstream of miR-146a exhibited reduced chromatin accessibility in SLE monocytes, correlating with disease activity (a metric called SLEDAI scores). Further analysis revealed that this enhancer’s dysfunction was mediated by diminished binding of the transcription factor CCAAT/enhancer binding protein α (C/EBPα), which is downregulated in SLE monocytes due to inflammatory cytokines like IFN-γ and IL-6.

To restore miR-146a expression, the team employed a dCas9-VP64 to target the 32.5 kb enhancer in peripheral blood mononuclear cells, isolated from SLE patients. This intervention successfully upregulated miR-146a and subsequently suppressed the hyperactive IFN pathway, as evidenced by reduced expression of interferon-stimulated genes such as *ISG15* and *OAS1*. Importantly, the approach demonstrated cell-type specificity for monocytes, with minimal off-target risks, highlighting the potential of enhancer-targeted epigenomic editing in SLE therapy.

Yet, for translational purposes, the development of an effective delivery system for monocytes and macrophages to treat SLE will be necessary. It will be also important to target other cell types, such as T and B cells, which use different enhancers for the therapy to be more effective.

Another very promising approach to treat autoimmune diseases is to induce regulatory T cells (Tregs) to the particular antigen, which triggers an autoimmune response. Tregs play a critical role in immune tolerance, and their function depends on the stable expression of the transcription factor *FOXP3*. However, in induced Tregs (iTregs), which are generated in vitro by TGF-β stimulation, *FOXP3* expression is often unstable, particularly under inflammatory conditions, not like in thymus-derived Tregs (tTregs), in which *FOXP3* expression is stable.

To address this, Okada et al. employed CRISPR-dCas9-based epigenome editing to modify the *Foxp3* locus in mouse primary T cells [128]. They designed two epigenetic editors: one was dCas9-TET1CD to induce DNA demethylation at the *Foxp3* enhancer, specifically at the conserved non-coding sequence 2 (CNS2), and the second was dCas9-p300CD to promote histone acetylation at the *Foxp3* promoter. While dCas9-TET1CD achieved partial demethylation of CNS2, it only weakly stabilized *Foxp3* expression in iTregs exposed to inflammatory cytokines. In contrast, dCas9-p300CD robustly enhanced *Foxp3* transcription, especially in the presence of TGF-β, including under inflammatory conditions, and improved the suppressive function of iTregs in vitro. Very interestingly in another study, a simple cocktail consisting of (1) sodium butyrate (pan-HDAC inhibitor), UNC0646 (a methyltransferase inhibitor), and vitamin C (TET enzyme cofactor) synergistically induced complete demethylation at the *Foxp3* CNS2 enhancer region, and, therefore, robust stabilization of the iTreg phenotype [129], but this approach is less gene-specific than with the usage of dCas-based editors.

Now, iTregs are mostly obtained ex vivo from CD4+ T cells via lentivirus-mediated Foxp3 transduction, so they all become tolerogenic [130]. It raises several issues. First, lentiviral transduction is associated with random integration into the genome, which may have undesired consequences, so it is probable that epigenomic editors can be a safer alternative, as no integration happens in this case. Second, by turning the entire polyclonal population of CD4+ Tregs towards the tolerogenic pathway we can halt the immune response to cancer cells, viruses, pathogenic bacteria and other agents normally destroyed, so the next step will be the creation of antigen-specific iTregs.

### 3.9. Acute Organ Injury and Fibrosis (In Vivo Studies)

Fibrosis is a pathological process defined by the excessive accumulation of extracellular matrix components, particularly collagen, in tissues in response to tissue injury or chronic inflammation. It is a consequence of the dysregulation of the healing process rather than a disease in itself. Fibrosis can occur in numerous organs, including the heart, lungs, liver, intestine, kidneys, and skin. This results in a disruption of the normal structure and function of these organs [131].

Xu et al. employed dCas9-TET3CD to facilitate demethylation of the antifibrotic genes *Rasal1* and *Klotho* in a mouse model of renal fibrosis. Rasal1 encodes a Ras-GTP inhibitor, and its promoter is hypermethylated in renal fibrosis. Consequently, the upregulation of *Rasal1* expression in renal fibroblasts prevented renal tissue from developing fibrosis. Furthermore, demethylation of the *Klotho* promoter, which encodes a transmembrane co-receptor of fibroblast growth factor-23, in epithelial cells has been demonstrated to reduce fibrosis by up to 25% [132]. It is noteworthy that the use of a high-fidelity dCas9 variant that also carries the Y450A, N497A, R661A, Q695A, and Q926A mutations (derived from SpCas9-HF4 nuclease [133]) has been shown to be effective. The high-fidelity editor demonstrated a greater efficacy in reducing fibrosis, achieving up to a 50% reduction compared to a less than 30% reduction with conventional dCas9. This is attributed to the fact that the high-fidelity editor does not activate the off-target genes *Anxa4* and *Nlrp5*, which have profibrotic activity. Furthermore, the authors observed enhanced expression of the *EYA1* and *LRFN2* genes, whose promoters are hypermethylated in fibrosis, in cell culture. However, the consequence of this activation in vivo remains to be elucidated [132].

A parallel study was devised with the objective of preventing acute kidney injury in patients undergoing chemotherapy with cisplatin, a common adverse effect of this agent. The gene for interleukin-10 (*IL-10*), which has anti-inflammatory properties, and *Klotho* were activated using the SAM system. The latter two components were delivered using AAV2/9, which was injected into the tail vein of mice that expressed the Cas9 protein. This resulted in a reduction in tubular necrosis and damage, as well as a notable increase in the survival rate of mice following the administration of a high dose of cisplatin [80].

It is therefore proposed that the use of epigenomic editors which activate antifibrotic genes, including *RASAL1*, *KL*, and *IL-10*, may represent a promising approach for the treatment of acute kidney injury and fibrosis.

**Figure 9:**
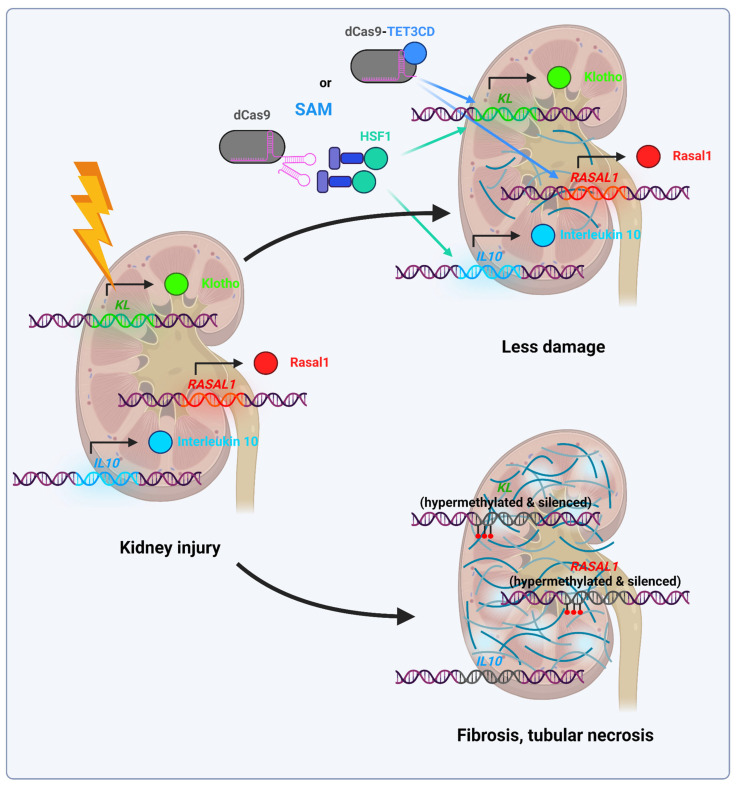
Epigenomic editors can be applied to minimize the consequences of acute organ (e.g., kidney) injury. For example, activation of *KL*, *RASAL1*, and *IL10* genes can decrease fibrosis and tubular necrosis after exposure to relevant factors. Created with BioRender.com.

Figure 9 showcases the role of epigenomic editors in mitigating consequences of acute organ (kidney) injury.

### 3.10. Normal and Accelerated Aging (In Vitro and In Vivo Studies)

One of the most ambitious potential applications of epigenome editors is the use of these technologies to combat the process of aging. Although the FDA has not yet recognized aging as a disease, which presents a challenge for the development of drugs, it is widely accepted among scientists that it is a risk factor for a range of diseases, including cardiovascular disease, cancer, and neurodegeneration. Accordingly, in order to combat these diseases, it would be prudent to target aging as the underlying cause that precipitates them [134].

It is well documented that the state of chromatin undergoes significant alterations with the progression of age. For example, there is a significant increase in euchromatin, but the formation of so-called senescence-associated heterochromatin foci (SAHF) also occurs, which is not a characteristic of young cells. This is due to a general decrease in DNA methylation and inactivation of histone tags such as H3K9me3 and H3K9me27. Additionally, DNA hypermethylation of individual promoters and global histone deacetylation occurs. This results in increased expression of mobile genetic elements, such as LINE1, which in turn gives rise to genomic instability, an elevated risk of cancer in old age, and inflammaging. In light of these alterations, epigenetic clocks have been devised to quantify the degree of epigenetic modification. A more detailed discussion of chromatin changes with aging can be found in other articles [135,136].

In their study, Liesenfelder et al. employed the use of dCas9-DNMT3A and CRISPRoff to achieve the methylation of the *PDE4C*, *FHL2*, *ELOVL2*, *KLF14*, and *TEAD1* genes in vitro, utilizing the HEK293T line and primary T lymphocytes as experimental models. The hypermethylation of these genes with age permitted the acceleration of the epigenomic clock by up to 10 years. Moreover, the methylation of these genes not only directly affects them but also indirectly affects neighboring genes and other genes that are hypermethylated with age and, thus, included in the epigenetic clock [137]. It may be possible to achieve rejuvenation, including in vivo tissue rejuvenation, through the demethylation of these or other genes with an age-dependent methylation pattern, or, conversely, through the methylation of genes that are hypomethylated during aging. It is imperative to consider the potential risks of tumor formation following the implementation of this procedure.

While the specifics of normal aging remain unclear and the development of new drugs is still a long way off, cases of pathological, accelerated aging, such as Hutchinson–Gilford progeria syndrome (HGPS), are recognized as diseases and require the urgent development of therapies. To this end, Kim et al. activated *Oct4* in HGPS mice using SAM. This resulted in partial rejuvenation at all levels, as evidenced by the following observations: (1) H3K9me3 levels increased while H4K20me3 levels decreased, (2) cellular senescence was initiated at an earlier stage, (3) DNA damage was elevated, (4) nuclear pores aggregated, (5) cell proliferation slowed, (6) vascular abnormalities, such as progerin accumulation and stiffness, were reduced, and (7) lifespan was prolonged. It is noteworthy that, despite OCT4’s role as an oncogene in numerous cancer types, the authors of this study did not observe an increase in tumor formation in the mice [138].

**Figure 10:**
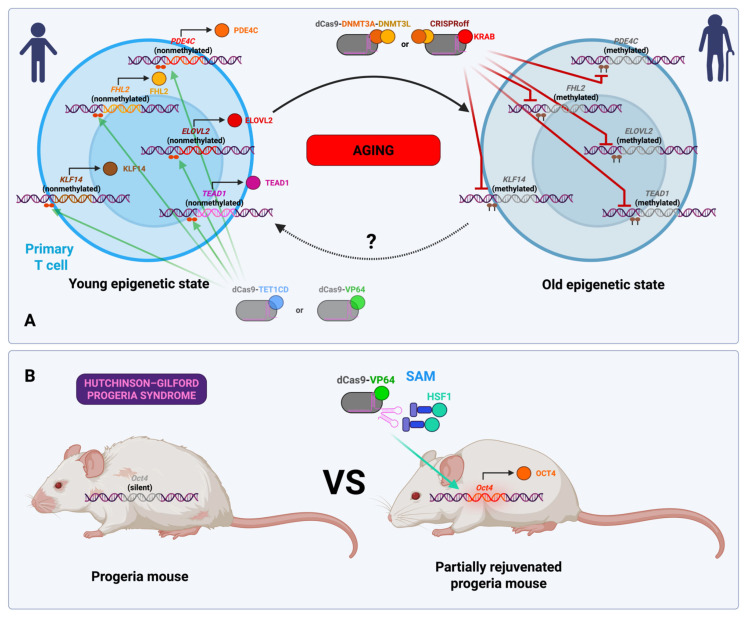
Epigenomic editors can be employed to slow or reverse aging. (**A**) methylation of several genes such as *PDE4C* can increase epigenetic age in some cell types; probably, demethylation of these genes in senescent cells can rejuvenate them (the reverse pathway is marked with a “?”). (**B**) activation of *Oct4* in HGPS mice can alleviate their symptoms. Created with BioRender.com.

Figure 10 shows how epigenomic editors can be used to fight aging.

### 3.11. Oncological Diseases (In Vitro Studies Only)

A further potential application of epigenome editors is the treatment of cancerous tumors [139]. This bears resemblance to conventional genome editing, in which the Cas protein is selectively delivered to tumor cells, where it effectively eliminates genes that are vital for their survival and reproduction. What, then, is the distinction between these two approaches and what is its purpose? The initial step will be an examination of the extant studies, which are currently limited in number.

Two fundamentally disparate approaches to their utilization are evident. The initial approach entails targeting the activating editor at tumor suppressor genes, thereby enhancing their expression. For example, dCas9-VP64, dCas9-VPR, and dCas9-p300 were employed to activate the expression of *MASPIN* and *REPRIMO* in a number of cell lines, including H157 lung cancer and MCF7 breast cancer cells. These editors demonstrated particular efficacy in the SAM modification, resulting in a 22,145-fold increase in *MASPIN* expression and a 680-fold increase in *REPRIMO* expression. The increased expression of *MASPIN*, an inhibitor of serine proteases, resulted in a reduction in proliferation and the induction of apoptosis. Meanwhile, the activation of *REPRIMO*, which acts through p53, causes cells to arrest at the G2 phase of the cell cycle [140]. The LbdCpf1-p300 construct was successfully employed to concurrently stimulate the expression of *MYOD*, a transcription factor linked to myogenic differentiation, and *IL1RN*, an interleukin-1 receptor antagonist, in U2OS (human osteosarcoma) and MCF7 cells [141]. In three other studies, the activation of oncosuppressor expression was achieved through demethylation of the relevant genes under the influence of TET1CD. In a study conducted on HeLa and MCF7 cell lines, dCas9-TET1CD was employed to activate the expression of *BRCA1*, a well-characterized oncosuppressor gene that plays a pivotal role in double-strand break repair and cell-cycle regulation. Mutations in this gene are frequently associated with hereditary forms of breast cancer. The activation of *BRCA1* resulted in the suppression of cancer cells [142]. The SunTag system, comprising (1) dCas9 fused to multiple GCN4 epitope repeats and (2) ScFv (GCN4-binding) fused to TET1CD, was employed to activate the *SARI* gene in a number of colorectal cancer cell lines. The demethylating effects of this editor on the *SARI* promoter resulted in the appropriate modulation of cancer cells, as *SARI* is a known inhibitor of proliferation and angiogenesis and an activator of apoptosis [143]. In a separate study, the identical SunTag system (dCas9-multiGCN4 + ScFv-TET1CD) was employed to induce the expression of *CARD9, SH3BP2*, and *CNKSR1* in A549 and 1–87 lung adenocarcinoma cell lines [144].

The second approach is the repression of genes that are important for tumor cell proliferation. For example, dCas9-KRAB was used to repress the *ΔNp63* gene in lung (EBC2) and esophageal (TE8 and KYSE70) squamous cell carcinoma cell lines. ΔNp63 is an isoform of *TP63*, which is proto-oncogenic and actively expressed in many cancers [145]. The *GRN* gene was repressed in Hep3B hepatoma cells by different editors, including dCas9-DNMT3a, dCas9-EZH2, and dCas9-KRAB. The GRN protein has been linked to the maintenance of cancer stem cells, the stimulation of cell growth, and the development of metastasis [146].

While this work has been conducted on cell lines, it is already possible to inquire as to the advantage of utilizing epigenomic editors in comparison to more conventional methods of cancer treatment, such as chemotherapy and radiation therapy, as well as genomic editing, which is also a promising approach in oncology today. Firstly, in contrast to conventional tumor therapies, both genomic and epigenomic editing can be less toxic to the human body due to the fact that CRISPR-based editors act on a single specific gene rather than the entire cell. Secondly, in contrast to genomic editing, epigenomic editors do not introduce double-strand breaks in DNA. Consequently, they are (1) considerably less toxic to healthy cells when expressed in them and (2) will not result in undesired genomic rearrangements in cancer cells, which can lead to unpredictable consequences. Third, in contrast to genomic editors, which can only knockout and therefore inactivate oncogenes, CRISPRa can be employed to activate the expression of oncosuppressor genes. Finally, in addition to inhibiting proliferation and simply killing cancer cells, a strategy to treat tumors by differentiating them into normal tissue is being developed, and epigenomic editors may prove to be a useful tool for this.

**Figure 11:**
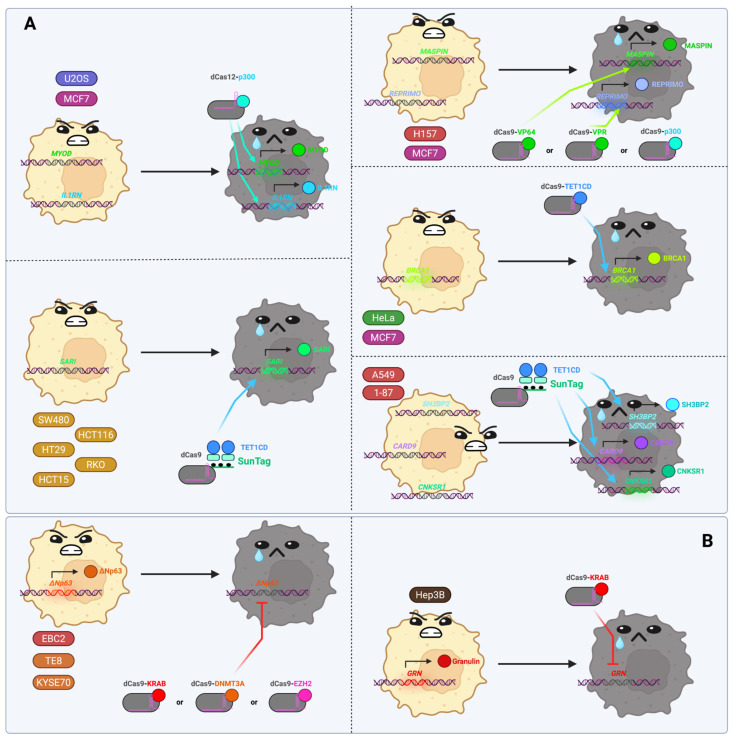
Epigenomic editors can be used to develop anticancer therapies. (**A**) activation of oncosuppressor genes (e.g., *MASPIN*, *REPRIMO*, *MYOD*, *IL1RN*, *BRCA1*, *SARI*, *SH3BP2*, *CARD9*, and *CNKSR1*) can inhibit the proliferation and activate apoptosis of cancer cell lines. (**B**) the same can be achieved via inhibiting of oncogenes (such as *ΔNp63* and *GRN*). Created with BioRender.com.

The use of epigenomic editors to address cancers is illustrated in Figure 11.

### 3.12. Viral Diseases (In Vitro, In Vivo Studies and Clinical Trials)

Another potential avenue for the application of epigenome editing is the development of therapies for viral diseases. To date, research has been conducted solely on the hepatitis B virus (HBV) and the human immunodeficiency virus (HIV). Two primary avenues of research have been pursued: the suppression of ongoing chronic (latent) infection, as demonstrated in the case of HBV, and the reactivation of the latent virus with the objective of further eradication of the infected cells. The latter approach, known as the “shock and kill” therapy, has been explored in the context of HIV. The following section will provide a more detailed examination of these strategies.

HBV is a DNA virus that attacks the liver. This infection can become chronic, which can contribute to cirrhosis and liver cancer. During this chronic infection, the viral genome stays in the latent form, being either integrated into the genome (intDNA), or staying as a separate 3.2 kb episome, which is called covalently closed circular DNA (cccDNA). Consequently, a number of approaches have been developed to reduce HBV activity by epigenomic editing [147,148].

Initially, an editor based on six zinc fingers and a KRAB domain, which targets the Enhancer I (EnhI) region, was constructed. EnhI controls the expression of numerous genes, including *HBx*, which encodes an X protein. This HBx protein is an important regulator of viral transcription, promoting processes such as infected cell survival and carcinogenesis. It activates as soon as the virus gets into the cell and suppresses complexes such as SMC5/6, which repress viral episomes inside the cell [149]. This results in a reduction in the levels of viral mRNAs, proteins, HBV genome replication, and particle production in both in vitro and in vivo models. In vitro models included the Hep3B and HepG2.2.15 cell lines, while in vivo models included transgenic mice into whose genome HBV was introduced [150,151]. Similar outcomes were observed in both in vitro and in vivo settings when a zinc finger and DNMT3A-based editor [110] were utilized, as well as when an ATF derived from TALEs and a KRAB repressor was employed [152]. Recently, Kostyushev et al. proposed an alternative approach: instead of repressing HBV genes, they designed a dSpCas9-p300-based CRISPRa system to activate genes related to the APOBEC/AID pathway, which is a natural mechanism to suppress viral infection. Transient activation of target genes, namely *A3A*, *A3B*, *A3G* and *AID*, increased their expression from ~4–8 to 800,000 times, which led to a reduction of cccDNA levels, even in HepG2-hNTCP and HepaRG-hNTCP cell lines, modeling established and severe chronic infection [153]. Finally, the US-based company Tune Therapeutics has recently started a phase 1b clinical trial of their drug Tune-401 (NCT06671093). Tune-401 utilizes a dCas9-TEMPO system, which combines dCas9 with effector domains (e.g., methyltransferases) to repress HBV via targeted methylation of a conserved “master controller” sequence [154]. The idea is to methylate CpG Island II, which is a key regulator of the HBx protein and HBV life cycle. Lipid nanoparticles are used for delivery. According to the information provided by the company in hepatocyte cell lines experiments, the proposed system achieves 90–95% repression of transcription from both cccDNA and intDNA, which is maintained for more than 1.5 years and is highly specific [155]. To our knowledge, it is the first large-scale clinical trial of the epigenomic editor-based drug on human patients.

HIV is the etiological agent of acquired immunodeficiency syndrome (AIDS), a severe disease that affects the immune system, primarily CD4+ T-helper cells, thereby rendering the body susceptible to infections. Currently, patients diagnosed with AIDS are required to adhere to antiretroviral therapy (ART) for the duration of their lives. This treatment blocks the action of the virus. However, HIV integrates its DNA into the host genome as a provirus, thereby establishing a latent infection. A latent infection is a significant concern as it can be reactivated when antiretroviral therapy (ART) is discontinued. This can contribute to chronic inflammation and a reduction in immune function. To address this, a strategy known as “shock and kill” therapy has been developed to eradicate the virus completely. This approach employs latency reversal agents (LRAs), which include histone deacetylase (HDAC) inhibitors, protein kinase C, and Toll-like receptor agonists. LRAs result in the activation of the transcription of the provirus embedded in the genome of CD4+ T cells. This enables the immune system to recognize and destroy infected cells, and the administration of ART during “shock and kill” therapy suppresses HIV replication and reduces the viral load in the body [156,157]. In 2016, three papers were published simultaneously, in which the authors employed CRISPRa constructs as a more selective form of LRA [158,159,160]. The studies employed dCas9-VP64, dCas9-VP160, SAM (consisting of dCas9-VP64, sgRNA, and MS2-p65-HSF1), and SunTag (with multi-GCN4 as epitope and ScFv-VP64 recruited). In all three papers, CRISPRa was targeted at the U3 sequence of the 5′-long terminal repeat (5′-LTR), as this region is a regulator of HIV transcription. The degree of transcription activation was found to be highly dependent on the specific position and type of editor. For instance, up to a 16-fold increase in transcription was observed with dCas9-VP64 and dCas9-VP160 [160], while up to an 11-fold increase was noted with SAM [158]. Similarly, a 6-fold increase was seen with SunTag [159]. All experiments were conducted in vitro on T-cell lines, including J-Lat, LChIT, C11, and others, which are models of HIV latency. Reporter genes were employed to assess transcription activation. Moreover, Bialek and colleagues observed the formation of complete viral particles following activation in J89 cells, indicating that HIV is fully activated by this method [158].

It is recommended that both approaches be taken into consideration. The suppression of infection may prove an effective strategy against DNA-containing viruses such as hepatitis B virus (HBV), herpes simplex virus (HSV), human papillomavirus (HPV), and adenovirus, among others. Perhaps more promising than the use of ATFs is the cutting of the virus genome using, for example, CRISPR/Cas. However, this approach may prove to be inefficiently effective, especially if the virus is embedded in the host cell genome. Furthermore, it may lead to undesired mutations and not result in the suppression of all virus genes. Accordingly, in certain instances, it may be prudent to contemplate the deployment of epigenome repressors as a means of regulating chronic viral infections. In regard to the utilization of CRISPRa for the “shock and kill” therapy of HIV, this strategy should supersede the currently employed LRAs due to its enhanced selectivity. It is important to note that other editors, such as representatives of the CRISPR/Cas type 6 family, including Cas13, should be employed to target RNA viruses. For example, in a recent study, the approach was successfully applied to suppress human enterovirus infection in the brain and hindlimb muscles of mice, which broadens the prospects in this field [161].

**Figure 12:**
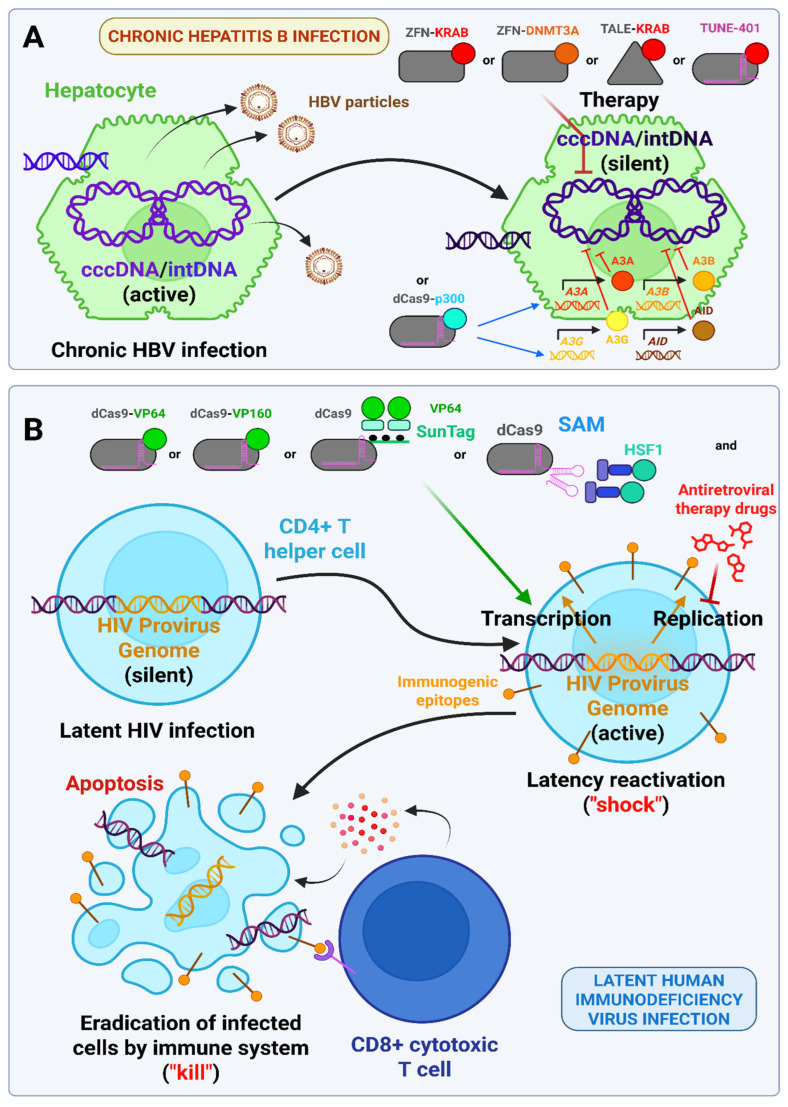
Epigenomic editors can be used to control viral diseases. (**A**) inhibition of the HBV genome can prevent liver cancer and improve the life of people with chronic hepatitis B infection. (**B**) activation of HIV genome with antiretroviral therapy use can be employed as an alternative “shock and kill” therapy for chronic AIDS. Created with BioRender.com.

Figure 12 highlights the employment of epigenomic editors in the treatment of virus-caused morbidities.

### 3.13. Regenerative Medicine and Cell-Based Therapies (In Vitro Studies)

**Figure 13:**
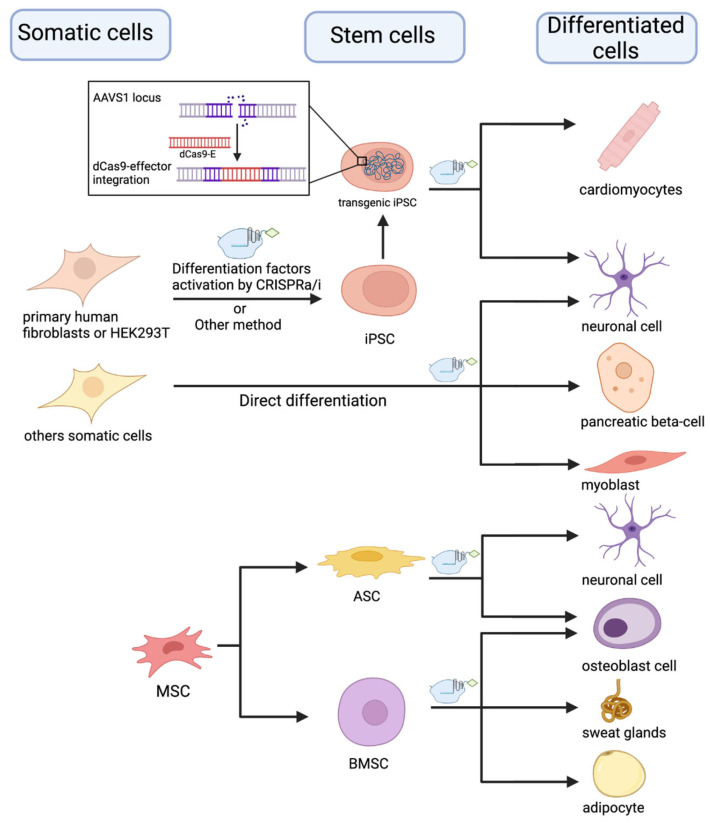
Cell differentiation based on CRISPR-dCas systems. dCas9-based systems are used to reprogram somatic cells into iPSCs and differentiate iPSCs into various cell types. dCas-based activators are also used to differentiate stem cells (ASC, BMSC) into various cell types. Created with BioRender.com.

The objective of regenerative medicine is to restore the functionality of organs and tissues that have been compromised by disease or injury. A significant area of focus within this field is cell therapy, where stem cells represent a promising source of replacement cells. Stem cells possess the capacity for multidirectional differentiation and self-renewal. There are multiple sources of stem cells, including embryonic (embryonic stem cells, ESC), adipose tissue (adipose-derived stem cells, ASC), bone marrow (bone marrow-derived mesenchymal stem cells, BMSC), blood (hematopoietic pluripotent stem cells, HSC), as well as artificial reprogramming of somatic cells to generate induced pluripotent stem cells (iPSCs). dCas-based systems can be used to trigger cell reprogramming and differentiation by precisely activating endogenous transcription factors [162]. This subsection will examine the application of dCas protein-based epigenome editing technology for regenerative medicine, with a particular focus on iPSC production and differentiation, as well as the reprogramming of other stem cells that can be used in transplantation, and, additionally, the direct reprogramming of cells within the body will be discussed. Figure 13 further illustrates this section.

The canonical reprogramming factors for iPSC generation include *OCT4* in combination with *SOX2*, *MYC*, *LIN28*, *NANOG*, and *KLF4*, with *KLF4* being an oncogenic factor. The initial efforts to generate iPSCs with CRISPR-based epigenome editing were not entirely successful. In one of the earliest studies, the activation of *OCT4*, *SOX2*, *KLF4*, *MYC*, and *LIN28* with dCas9-VP64 via lentiviral transduction did not result in the effective generation of iPSCs [163]. Balboa D. et al. [164] demonstrated the capacity to stimulate the expression of differentiation genes through the use of VP192 in HEK293 cells, employing an electroporation technique with plasmids. However, the activation of all necessary factors, including *KLF4* and *LIN28*, and the subsequent achievement of iPSC formation were not feasible. Nevertheless, pluripotency was maintained up to 10 divisions, and the cells exhibited partial reprogramming into endoderm and pancreatic cells. The use of a dCas9VPH-based CRISPRa approach, targeting only endogenous promoters of key differentiation genes, was sufficient to successfully reprogram neuroepithelial stem cells into iPSCs. However, this approach was not effective in reprogramming epithelial stem cells (HEK293) [165]. Subsequently, combination approaches that target additional regions have been developed with the objective of enhancing the efficiency of successful reprogramming. For example, additional gRNA targeting of the EEA motif combined with p53 repression by shRNA has been demonstrated to induce genomic instability [165], while targeting the miR-302/367 cluster together with the EEA motif has been shown to enhance reprogramming efficiency while reducing the background of unreprogrammed cells [162]. In a recent study, Lee et al. [166] employed a cell delivery method utilizing magnetic chitosan nanoparticles sealed with peptides containing immobilized dCas9-VPR-srRNA complexes, which proved to be an effective approach for reprogramming HEK293T cells into iPSCs. Abujarour R. et al. employed a SunTag-based CRISPRa-SM system and inhibitors of GSK3β (CHIR99021), MEK/ERK (PD0325901), TGF-β (SB431542), and Rho-kinase (Thiazovivin) for reprogramming to produce human iPSCs with typical morphology, without activating *KLF4*, the Alu locus, and using additional exogenous RNAs [167]. Consequently, CRISPR-based tools have been effectively utilized to derive iPSCs, and they may also be employed to obtain them for therapeutic applications.

The development of CRISPR-dCas-based gene regulation in iPSCs, including methods for further differentiation, is also a current area of active research. The CRISPRa and CRISPRi approaches have been successfully used to differentiate iPSCs generated via conventional methods into neuronal cells [45,168,169] and cardiomyocytes [170,171]. For instance, iPSC-derived cardiomyocytes may prove useful in cell therapy for the treatment of myocardial infarction. In a recent study, Qiao and colleagues employed CRISPRa to regulate the expression of an oncomarker gene, which is essential for the differentiation of cardiomyocytes from iPSCs [172]. The primary methodology for achieving such differentiation is the creation of stable transgenic iPSC cell lines through the integration of dCas9, which is fused to transcription factors, into regions of the genome that are deemed safe. This approach is preferable to random integration of the transgene, which can result in low levels of expression, or instability [173]. Consequently, a number of studies have successfully integrated dCas protein-based constructs into safe loci, thereby demonstrating the ability of such iPSCs to differentiate [174,175]. Furthermore, methodologies are being devised to enhance the efficiency of gRNA delivery, as cellular differentiation necessitates the simultaneous targeting of multiple genes [176].

Furthermore, genomic screens facilitate the identification of genes that play a pivotal role in both cell reprogramming and pathological processes of already-reprogrammed cells, thereby expanding the potential for cellular differentiation [174,177]. Nevertheless, despite the successful establishment of transgenic lines and the high level of CRISPRa/i expression, only one recent study has analyzed dCas9-editors expression after differentiation. This study demonstrated a significant decrease in dCas9-VPR levels between day 2 and day 5 of differentiation into cardiomyocytes and cardiac endothelial cells. This finding highlights the importance of verifying cell line stability for each application [171].

Additionally, CRISPR-based activators have demonstrated efficacy in differentiating mesenchymal stem cells into adipocytes [178]. The activation of neurotrophic factors in adipose stem cells resulted in the regeneration of peripheral neurons, as evidenced by the regeneration of the sciatic nerve following the implantation of a sheet of transgenic adipose stem cells (ASCs) into the mouse hind limb [179]. The implantation of BM-MSC with a dCas9-VP64 construct, integrated by lentiviral transduction, into a burn wound in mice resulted in the repair and healing of the injury, as evidenced by the regeneration of sweat glands [180]. The endogenous overexpression of *PDGFR-β* in ASCs using CRISPR-SAM also resulted in enhanced wound healing when the cells were transplanted into diabetic mice [181]. Moreover, VP64, SunTag and SAM activators have been demonstrated to facilitate the reprogramming of mesenchymal cells into osteogenic cells [182]. The differentiation of osteogenic and chondrogenic cells represents a crucial objective of regenerative medicine, offering a potential way for the treatment of bone and cartilage defects in adults that are challenging to repair rapidly. Bone healing can occur via two distinct pathways: intramembranous and endochondral. In the first case, progenitor cells differentiate into osteoblasts, while, in the latter, progenitors first transform into chondrocytes, creating a cartilaginous matrix that subsequently ossifies. In a recent study, Nguyen et al. [57] employed a cleaved LbdCas12a system in conjunction with p65-HSF1 to activate the long non-coding RNA H19, which has been demonstrated to stimulate osteogenesis and chondrogenesis. Notably, the partitioned system demonstrated enhanced efficiency relative to the full-length LbdCas12a protein. The induction of H19 in bone marrow stem cells (BMSCs) prior to implantation into 6-week-old rats in 6 mm carvarial defects resulted in the suppression of adipogenesis and an improvement in chondrogenic differentiation. Subsequently, the same research group enhanced the chondrogenic differentiation of ASCs by activating not only the *H19* gene but also the *SOX5* and *SOX6* genes. This resulted in an improvement in the efficiency of skull vault bone regeneration in rats [58]. ASCs offer a distinct advantage for reprogramming due to their relatively less painful isolation from the body, which allows for a greater yield of stem cells compared to BMSCs. Other studies have indicated that maintaining the stability of non-terminalized chondrocytes represents a significant challenge. Shi et al. [183] elucidated the molecular mechanisms of chondrogenesis by activating deubiquitinase UCHL1 with SadCas9-VPR-based CRISPRa, which stabilized chondrocytes and promoted the localization of HIF-1α, a factor that activates the expression of the chondrogenic factor *SOX9*. In a study on cartilage repair in osteoarthritis [184], CRISPRa/i were used to activate *SOX9* and repress a transcription factor involved in the activation of tumor necrosis factor *RelA*. The results demonstrated that this approach effectively enhanced the differentiation of mesenchymal stem cells (MSCs), and the intra-articular administration of these cells led to a reduction in cartilage degradation in a mouse model of osteoarthritis. Another significant challenge in regenerative medicine is liver fibrosis, which arises when hepatic stellate cells (HSCs) undergo a transformation into collagen-secreting myofibroblasts, leading to epithelial–mesenchymal transition (EMT). In a study conducted by Adisasmita et al., dCas9-KRAB-based CRISPRi was employed to suppress *SOX9* gene expression in chemically induced human hepatocyte progenitor cells (mCdHs) derived from human primary hepatocytes (hPHs) with the objective of treating liver fibrosis. The findings of this study identified a role for SOX9 in liver regeneration [185]. Additionally, EMT plays a pivotal role in trophoblast differentiation during the initial stages of placental development. Disruption of this process is linked to complications associated with pregnancy. Perez-Garcia et al. [186] demonstrated the involvement of the BAP1 gene in trophoblast invasion through the use of Cas9 knockout approaches with nuclease activity, as well as CRISPR-SAM to overexpress the gene. It has been demonstrated that gene knockout can initiate EMT in mouse trophoblast stem cells (mTSC). Furthermore, *BAP1* activation has been shown to slow cell proliferation, suppress differentiation into giant trophoblastic cells (TGC), and reduce trophoblast invasion. It can be postulated that the utilization of the CRISPRi approach to inhibit gene expression may also prove to be a fruitful avenue of investigation, particularly in the context of pregnancy-related complications.

CRISPR-dCas-based regulation also has the potential to reprogram somatic cells. For example, Liu et al. demonstrated the potential of using dCas9-Tet1 to reprogram mouse embryonic fibroblast (MEF) cells into myoblasts [187]. Another example is the reprogramming of mouse embryonic fibroblasts into induced neuronal cells [188]. Furthermore, the CRISPRa-based enhancement of endogenous ectodermal dysplasia ectodysplasin (EDA) gene expression in keratinocyte cells and their successful reprogramming into sweat gland cells has also been reported, which is an important aspect of wound-healing therapy [189]. An equally significant area of focus in regenerative medicine is the generation of beta cells for insulin production. Thus far, attempts to reprogram HEK293 cells into pancreatic beta cells have been documented, as have been attempts to switch alpha cells to beta cells using CRISPRa or CRISPRi systems [190,191,192,193].

Cell therapy technologies typically entail the transplantation of cells obtained in vitro. However, direct reprogramming of cells in vivo, which obviates the need for transplantation, represents a promising avenue for the treatment of various diseases. Nevertheless, it is essential to develop methodologies that are independent of ectopic expression of differentiation factors, which can be achieved through the use of CRISPR-dCas-based epigenetic editors. For example, in a mouse model of Parkinson’s disease, delivery of an adenovirus construct by CRISPR-SAM via injection into the brain resulted in successful reprogramming of astrocytes into functional neurons through the activation of differentiation factors *Ascl1*, *Lmx1a*, and *Nr4a2* [106]. Furthermore, CRISPR-SAM was employed to stimulate the expression of multiple *IGF1* gene isoforms in human and mouse myoblasts in vitro, which facilitated myogenic differentiation and may prove beneficial for muscle atrophy therapy [194]. Furthermore, a CRISPRa-based system comprising dCas9-VP64 delivery via exosomes into hepatic stellate cells (HSC) was employed to induce expression of the hepatocyte differentiation factor HNF4a, thereby facilitating their reprogramming into liver cells. This approach was demonstrated in both in vitro and in vivo settings by introducing a plasmid construct within exosomes into the mouse tail vein, offering a novel therapeutic approach to treat liver fibrosis [195]. In a 2020 study, Joo and colleagues employed the CRISPRa system in mouse corneal endothelial cells, delivering dCas9-VPR via electroporation in vivo. This approach led to the regeneration of these cells after cryopreservation [196].

It is therefore evident that epigenetic editing represents a significant and promising avenue of research in the field of regenerative medicine. To date, tools based on the CRISPR system have enabled the generation of induced pluripotent stem cells (iPSCs), their subsequent differentiation into various types of somatic cells, and the reprogramming of other types of stem cells for ex vivo therapy through implantation of the obtained cells (Figure 13). This has also facilitated the development of techniques for the direct treatment of diseases without the need for the transplantation of the cells into the body.

Immunotherapy against hematological malignancies, based on the adoptive transfer of T cells carrying a chimeric antigen receptor (CAR), showed its tremendous efficiency over the last decade. Autologous T cells are getting genetically modified with CAR which is able to target T cells on a particular antigen and activate its cytotoxic function against tumor cells carrying this antigen [197,198]. A chimeric antigen receptor, approved for clinical practice, consists of the antigen recognition extracellular antigen binding domain, transmembrane domain and intracellular costimulatory and signaling domains. Each structural unit of the receptor provides CAR-T cells with important functional properties, such as the ability to proliferate and persist in vivo or prevent the development of exhaustion [199]. But, it not only the structure of the receptor that influences the anti-tumor efficacy of CAR-T cells, but also the tumor cell features, for example, the presence of antigens on the cell surface. One of the main problems associated with this is the complete loss of the target antigen by tumor cells [200,201,202]. Thus, despite the success of CAR-T-cell therapy, there exist several opportunities to improve its effectiveness and safety using the dCas systems.

Programmed cell death protein (PD-1), while expressing in activated T cells, lowers the efficacy of the CAR-T-cell therapy by decreased persistence of the T cells [203]. Yang et al. created CAR-T cells (RB-340-1) that combine the antigen binding function with the CRISPRi system to suppress *PD1* gene expression. Two lentiviral constructs were used to transduce T cells: one of them carried the CAR with TEV (tobacco etch virus) protease and sgRNA, the second one encoded a linker for the activation of T cells (LAT), fused to dCas9-KRAB via a TEV-cleavable sequence (TCS). After the activation of CAR, two constructs are close enough for protease to release the CRISPRi system. In vivo experiments revealed the prolonged persistence and increased efficacy of CAR-T cells received with such an approach [204]. The analogous scheme was used to activate IL-2 expression by dCas-VP64 in order to induce the IL-2-promoted proliferation of T cells [205,206].

In the field of cell therapy, CRISPRa/i technologies can also be useful from a research point of view. For instance, Lee et al. used CRISPRa (dCas-VP64) screens to reveal the key genes of NK- and T-cell evasion mechanisms [207]. A bidirectional CRISPRa/i system, based on doxycycline (dox)-inducible dSaCas9-VPR and SpdCas9-KRAB, ZNF10 or KOX1 domain to affect two different loci simultaneously in the same cell, was performed in order to study the interaction of two hematopoietic lineage transcription factors, SPI1 and GATA1 and their coregulation. CRISPRi machinery was also used to investigate the mechanisms of enhancer-mediated *IL2* gene regulation in T cells [208]. Ramkumar et al. applied several approaches based on CRISPRi and CRISPRa screens to identify pathways of B-cell maturation antigen expression and identify mechanisms of myeloma to BCMA-targeted CAR-T-cell response [209]. Such studies can provide scientists with new insights that are applicable to the development of the next generations of cell therapies.

## 4. Animal Biotechnology Applications

There are several applications which should be highlighted in the field of animal biotechnology. This includes animal husbandry, where biotechnology is used to improve the quantity and quality of animal products (not only food but also certain materials such as silk and wool), facilitate animal breeding and improve animal health and quality of life. Other fields include xenotransplantology and the creation of model animals for biomedicine. The creation of GMOs (transgenic animals) is heavily regulated in many countries. As discussed earlier, some scenarios of epigenetic editing may require only transient action of the editors (through RNPs or transient expression) without the incorporation of exogenous DNA into the animal’s genome. In the latter case, in certain jurisdictions the animal may not be considered as a GMO—which may be an advantage of epigenetic editing over genome editing [210]. However, sometimes a stable expression of the epigenomic editor is necessary to achieve the desired result, and in such cases the integration of genes related to epigenomic editor is inevitable, i.e., the animal will become transgenic [79].

There are many ways to deliver an epigenomic editor into an animal cell. Such methods include the following: implantation of pre-epigenetic edited cells into the animal organism; transduction using AAV (adeno-associated virus); purification and targeted delivery of ribonucleoprotein complexes to the desired organs and tissues (e.g., as part of complexes with metals and lipid nanoparticles) [211].

Nowadays, epigenetic editing in animal biotechnology is just starting to develop. In the field of animal husbandry, we can highlight the work on shifting the sex balance in mice towards females by introducing a dCas9-based epigenomic repressor (dCas9-KRAB) into the Y-chromosome, which inhibits the expression of the gene responsible for spermatid maturation [212]; as a result, transgenic males produce sperm containing mainly X-chromosome, and the females born from such sperm will not be transgenic—such a shift in the balance of sexes is useful in animal breeding, where the offspring of unwanted sex (males), for example, in cows and chickens, are euthanized. To our knowledge, the rest of the work in the field of animal biotechnology is being done mostly in vitro—for example, attempts are being made to create sheep germ cells from Leydig cells by increasing the expression of *Bmp4*, *Dazl*, *Nanos3* and *Sycp2* genes with the help of dCas9-SAM [213]. In a bovine mammary gland mastitis cell model, demethylation directed by the dCas9-C-Tet1-SgRNA 2.0 system led to improved synthesis of milk proteins [214]. Potential areas of animal biotechnology development using CRISPR-based epigenomic editors include the following: (1) creation of livestock with suppressed expression of myostatin (transgenic pigs with this trait were successfully created with Cas9 genome editing) [215], (2) creation of animals with increased expression of growth hormone (GH)/insulin-like growth factor (IGF) which would increase the muscle mass of the animal and have a positive effect on nutritional value (for example, transgenic pigs with *IGF1* knock-in were previously created via the integration of pig-derived *IGF1* gene into a *pRosa26* locus [216], transgenic coho salmon (*Oncorhynchus kisutch*) and several other members of *Oncorhynchus* genus were created via insertion of gene construct OnMTGH1, which contains *GH* gene from *Oncorhynchus nerka* [217]); (3) creation of pigs with suppressed expression of alpha-1,3-galactosyltransferase (*Ggta1*), which would result in the absence of the antigen recognized by the human immune system on the surfaces of its cells and provide organs for xenotransplantology (previously, permanent knockout of this gene was achieved via usage of gene trap vector pGalGT [218]); (4) sex change in fish through regulation of androgen/estrogen expression [219,220] (fish can be simply fed hormones with food, but it may be dangerous for the environment and, consequently, to animals and humans [221,222]).

To summarize, epigenomic editing in the field of animal biotechnology is an approach currently under development, so, unfortunately, there are not many studies to review. But it probably will become more prominent in the coming years, since there are certain potential applications and advantages to that technology in comparison with ordinary genetic editing.

## 5. Applications of Epigenomic Editors in Agrobiotechnology

### 5.1. Peculiarities of Epigenetic Regulation and Engineering in Plants

Epigenetics plays a crucial role in plant life, influencing how plants respond to their environment without altering their DNA sequence [223,224]. Epigenetic changes, also known as epialleles, are often heritable in plants, meaning that the adaptations can be passed onto future generations, providing long-term evolutionary benefits [225].

The *FWA* gene is a classic example of heritable epigenetic regulation in plants, where stable DNA methylation silences expression in wild-type plants, while loss of methylation leads to a late-flowering phenotype that is inherited across generations without changes in DNA sequence [226]. Remarkably, both methylated (silenced) and unmethylated (active) *FWA* epialleles behave as stable Mendelian traits, persisting in natural Arabidopsis populations for many years, demonstrating that epigenetic marks can be as enduring and heritable as genetic mutations.

Epigenetic states in plants may be altered by external stimuli and then maintained during plant growth and, in certain cases, even be passed to future generations (see below). One well-known example of such a phenomenon is vernalization in *Arabidopsis Thaliana* [227]. Vernalization is the process by which plants require exposure to cold temperatures to flower. This adaptation ensures that flowering occurs in the spring rather than in winter. In *Arabidopsis*, the gene FLOWERING LOCUS C (*FLC*) acts as a floral repressor, preventing flowering. During vernalization, prolonged cold temperatures lead to the epigenetic silencing of the *FLC* gene through chromatin modifications, particularly through the methylation of histone proteins associated with the FLC locus. This silencing reduces *FLC* expression and allows the plant to initiate flowering when temperatures rise [228]. The silenced state of the *FLC* gene can be stably maintained and passed on through cell divisions, allowing the plant to “remember” that it has experienced cold and thus remain ready to flower in warmer conditions [229]. Despite the fact that *FLC* epialleles are not normally inherited through sexual reproduction [230], it has been shown that mutations in the *DDM1* (decreased DNA methylation) locus, can render such epimutations heritable [231]. The ability to create new heritable epialleles opens new avenues for enhancing crop traits and improving agricultural practices through epigenetic manipulation.

Despite their significant morphological differences and evolutionary divergence, plants and animals share remarkable similarities in genome and epigenome organization, particularly between plants and mammals. For example, the genome size, complexity, and ratio of heterochromatin to euchromatin in seed plants are often comparable to those in mammals. Both groups utilize DNA methylation and histone post-translational modifications (PTMs) for gene regulation. However, plant epigenetics is distinct from that of animals due to unique developmental traits, such as greater environmental plasticity and the ability to continuously form new organs throughout their lifespan [232]. This allows plants to adapt to changing environmental conditions through epigenetic modifications, facilitating rapid phenotypic changes in response to stressors.

Plants possess distinct transcriptional effectors, such as ethylene response factors (ERFs), which play a vital role in modulating gene expression. ERFs serve as key regulators of ethylene signaling, particularly in mediating plant defense responses to both abiotic and biotic stresses [233]. Notably, the ERF/EREBP family of transcription factors is especially significant, as these regulators feature domains with motifs that are not specifically designated for DNA binding [234]. Within this family, the SRDX motif, derived from the ERF-related amphiphilic repressor domain (EAR), has been identified as conferring repressive activities [235]. Additionally, another transcriptional regulator within the ERF family, characterized by the EDLL motif, functions as a potent activation domain [236].

In animals and plants CpGs are methylated de novo by the proteins of DMNT (DNA methyltransferase) family in mammals and by the orthologue of DNMT1—MET1 (Methyltransferase 1) in plant cells. However, there are other sites in plants that are targeted by MTs. CMT3 (Chromomethylase 3) is responsible for CHG site methylation (H stands for A/T/G), CMT2 methylates CHH sites. An alternative way to methylate these sites is carried out by DRM2 (Domains rearranged methyltransferase), which is directed to its targets via non-coding RNA molecules (RdDM—RNA-dependent DNA Methylation) [237]. In plants, DNA-glycosylases ROS1 (Repressor of silencing 1) and DME (DEMETER) catalyze base excision repair (BER) of 5mC demethylation without intermediates [238]. DME’s paralogs, DML2 and DML3, also contribute to methylation dynamics in various contexts [239]. Histone modifications in plants share significant similarities with those in animals. For instance, the dynamics of histone modifications, including the presence of specific marks like H3K27me3 associated with gene silencing, are conserved across plant and animal systems, indicating a fundamental role in transcriptional regulation [240].

In plants, DNA methylation and histone modifications play crucial roles in responding to external stimuli [241]. For instance, in *Zea mays*, DNA methylation in the *ZmNAC111* promoter represses *ZmNAC111* expression, resulting in increased drought sensitivity [242]. Conversely, in *Gossypium hirsutum*, improved drought tolerance has been linked to decreased levels of H3K9ac (a histone acetylation mark) in the promoter region of the *GhWRKY33* gene, regulated by the histone deacetylase GhHDT4D [243]. In rice cultivars subjected to salt stress, significant changes in DNA methylation patterns were observed, leading to hypermethylation and hypomethylation of specific genomic regions, which, in turn, altered gene expression patterns to enhance salt tolerance [244]. Additionally, in *Lycopersicum esculentum* (tomato), inoculation with the pathogen Botrytis cinerea resulted in the upregulation of genes involved in the oxylipin pathway and stress-responsive transcription factors like WRKY75, coinciding with increased levels of histone marks H3K4me3 and H3K9ac, highlighting the role of histone modifications in defense responses [245]. Thus, epigenetics is crucial for crop plants in relation to stress, highlighting the need for effective management, and CRISPR provides us with the tools to achieve this by enabling precise modifications to enhance stress resilience.

The delivery methods for dCas9 components in plants differ significantly from those used in animal systems due to the unique challenges posed by the presence of the plant cell wall, which acts as a barrier to the delivery of biomolecules. Additionally, while animal cells can be easily cultured and manipulated in vitro, plant cells require regeneration from tissues or protoplasts, making the delivery process more complex and time-consuming [246,247].

Genetic transformation in plants can be achieved through various DNA delivery methods for plant transformation, targeting protoplasts, individual cells, tissues, or whole plants [248]. For protoplasts, techniques such as polyethylene glycol (PEG)-induced DNA uptake and electroporation are commonly employed. When focusing on individual cells, microinjection is a precise method that enables targeted delivery of DNA into single cells, offering high transformation efficiency, but it is a slow process that demands significant technical skill. These methods for facilitating DNA uptake are straightforward and cost-effective. However, it requires labor-intensive protocols for the subsequent regeneration of viable plants and is limited to protoplasts or cells. Additionally, these means can lead to chimeric plants, complicating the analysis of transgenic traits [249]. For tissue transformation, Agrobacterium-mediated methods are widely used, leveraging the natural ability of Agrobacterium to transfer DNA into plant cells [250]. This method can achieve high transformation rates but typically requires a tissue culture phase, which also can be time-consuming and complex. In contrast, in planta transformation methods allow for the direct introduction of DNA into intact plants, bypassing the need for tissue culture. In planta approaches include viral vectors and Agrobacterium-mediated methods. Viral vectors utilize plant-infecting RNA and DNA viruses to deliver genes by integrating them into the viral genome, allowing for high levels of expression; however, this typically results in transient expression [251]. In contrast, Agrobacterium-mediated transformation can lead to stable integration and the production of transgenic plants. These approaches can be faster and more straightforward, but may result in lower transformation efficiency compared to other methods. Overall, the choice of method depends on the desired outcome and the specific characteristics of the plant being transformed.

Stable and transient expression are two distinct approaches used in plant biotechnology for gene delivery and protein production. Stable expression involves the integration of genetic material into the plant genome, resulting in heritable changes. This method is essential for creating transgenic plants that exhibit permanently desired traits. The process of stable transformation is complex and time-consuming, often requiring tissue culture techniques and selection of transformed plants, which can take several months to achieve [252,253]. In contrast, transient expression refers to the temporary introduction of dCas9 components into plant cells, allowing for rapid protein production without the need for stable integration into the plant genome. This method is particularly useful for producing proteins quickly and is advantageous for epigenetic editing studies, as it can yield high levels of protein in a short time frame, typically within days [254,255], and provides the means to function without permanently altering the plant’s genetic makeup. Consequently, this approach is essential for developing non-GMO plants [256].

DNA-free delivery methods in plants offer significant advantages over transient plasmid transformation. Unlike plasmid-mediated transformation, which risks random integration of foreign DNA into the host genome, techniques such as ribonucleoprotein (RNP) transfection enable the delivery of CRISPR components without altering the plant’s genetic makeup [234,257,258]. This not only minimizes off-target effects and enhances editing efficiency but also aligns with regulatory frameworks that favor non-GMO products, thereby facilitating societal acceptance. Despite the implementation of DNA-free, transgene-free delivery methods for Cas constructs in plants, there is currently a lack of studies focusing on the delivery of dCas constructs via these innovative techniques.

Plant epigenetics presents unique opportunities for engineering heritable and reversible gene expression changes. The distinct mechanisms of DNA methylation and histone modifications, coupled with environmental plasticity and specific regulatory proteins, position plants as highly amenable systems for epigenome editing strategies aimed at trait improvement.

### 5.2. Perspectives of Epigenomic Editing in Plants

In initial efforts at epigenetic editing in plants, individual non-catalytic domains, such as VP64, TAL, and EDLL, were fused to dCas9 for gene activation [259,260], while BRD and SDRX were employed for repression [259,261]. Subsequently, researchers began combining multiple effector domains in tandem, exemplified by TV (6xTAL–VP128) and VPR (VP64-P65-RTA), to enhance gene activation effects or 3xSRDX for gene repression [45,260,262]. A pioneering example of using a catalytic domain in plants was the fusion of the P300 acetyltransferase to dCas9, targeting the *AREB1/ABF2* gene in Arabidopsis, which is crucial for drought resistance [263].

The development of Synergistic Activation Mediator (SAM), scaffolding RNAs (scRNA), and SunTag strategies facilitated the recruitment of multiple regulatory domains, including catalytic domains, significantly improving the efficiency of epigenome editing [50,51,264]. CRISPR-Act 2.0 and CRISPR-Act 3.0, which integrated previous technologies with SunTag, were also implemented in plants [265,266]. Specifically for plant applications, the MoonTag system was developed, demonstrating superior activation levels compared to SunTag, stable expression in transgenic plants, and effectiveness across various plant species [267].

Moreover, novel programmable effector domains have been engineered for plants, expanding the possibilities for epigenome manipulation [268,269]. Researchers have also explored the use of dCas12a, dCas12b, and hypercompact Cas12j2 (CasΦ) for epigenetic editing in plants, offering enhanced delivery, targeting, and editing capabilities [270,271,272]. While several studies have employed knock-in and knockout approaches to regulatory elements to modulate gene expression [273,274], these methods are not classified as epigenetic editing in this review.

**Figure 14:**
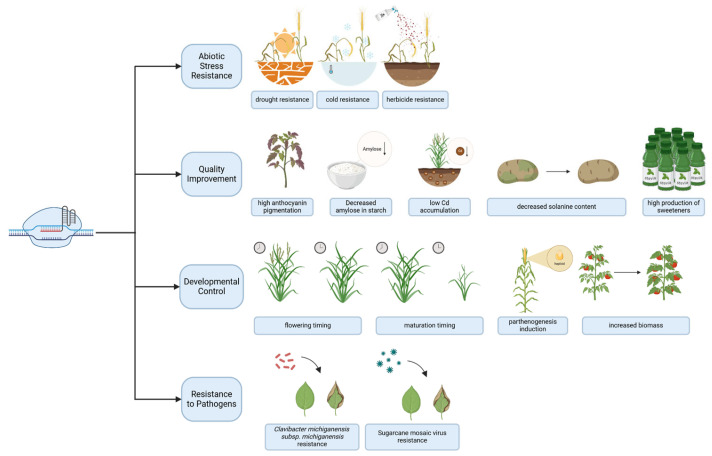
Overview of epigenome editing applications in agrobiotechnology. dCas9-based systems are used for targeted gene regulation in plants. Key applications include abiotic stress resistance, quality improvement, developmental control, and resistance to pathogens.

Epigenome editing in plants has emerged as a promising strategy to enhance various traits critical for agricultural sustainability and productivity. Figure 14 illustrates major agricultural applications of epigenome editing in plants. One of the key applications is in improving abiotic stress resistance. To improve drought tolerance in *Arabidopsis*, researchers developed a construct that fuses dCas9 with a histone acetyltransferase to activate the *AREB1* gene [263]. This modification resulted in transgenic plants exhibiting higher survival rates and chlorophyll content under drought stress compared to controls. Additionally, other studies demonstrated that activating *AREB-1* and *AVP1* genes led to increased relative water content and ion accumulation, further enhancing drought resistance in these plants [275,276]. dCas9-TV was used to activate the drought-related gene *GhTLP19* and *GhTULP34*, which inhibits stomatal closure in response to osmotic stress in Cotton [277]. This allows plants to better withstand periods of low water availability, ultimately leading to improved yields in arid regions. Herbicide tolerance is a key functional trait in genetically modified crops. The optimized dCas9-TV system was also employed to activate the *GhEPSPS* gene, resulting in the production of glyphosate-tolerant cotton [277]. Both the transformed plants and their progeny demonstrated resistance to glyphosate. The dCas9-TV system was used to achieve highly efficient activation of the cold-responsive transcription factor gene *CBF4* in grape [278]. In comparison to wild-type plants, the CBF4-activated plants showed reduced electrolyte leakage following cold treatment.

Another significant area of application is the increase in biomass. Epigenetic modifications can be employed to regulate growth-related genes, promoting enhanced growth rates and overall biomass accumulation. For example, the *AVP1* gene activation leads to increased leaf number and leaf size [276]. The activation of the *SlWRKY29* gene in Tomato using the CRISPRa approach enhanced embryo induction and maturation [279]. By fine-tuning the epigenetic landscape, plants can be optimized for maximum growth potential.

Quality improvement is also a critical focus of epigenome editing. By modifying the expression of genes involved in metabolic pathways, researchers can enhance the nutritional content and flavor profiles of crops. For instance, CRISPRa-mediated increased production of anthocyanin pigment 1 (PAP1) induces purple phenotypes with high anthocyanin accumulation in *Arabidopsis* [265,276]. Anthocyanins are responsible for the diverse aesthetic coloration in various plant organs and provide health benefits. Transcriptional activation of the UDP-glucose flavonoid glycosyltransferases (*UFGT*) gene in grape cells also promoted anthocyanin biosynthesis [278]. The activation of *GhPAPID* resulted in a distinct red/purple phenotype in cotton [277]. In the future, one can employ the dCas9 system to modify the color of cotton fibers, offering a promising approach for developing naturally colored cotton. Contrastingly, increased flavonoid production was caused by the repression of the cinnamate4-hydroxylase (*NtC4H*) gene using CRISPRi [280]. CRISPR-Cas12j2 (CasΦ) was fused with the catalytic domains of DNMT3A and DNMT3L to decrease the expression of granule-Bound Starch Synthase 1 (*OsGBSS1*) in rice cells. This was achieved through targeted DNA methylation of the gene promoter, influencing the amylose/amylopectin ratio in starch. Rice with increased amylopectin content typically has a softer texture and stickier consistency when cooked, making it desirable for various culinary uses [272]. CRISPR-dCas9-TET1 was employed to regulate the demethylation of the *OsCTF* 3′ UTR in rice, enhancing the expression of the *OsCTF* gene to reduce cadmium accumulation [281]. Cadmium (Cd), a heavy metal that contaminates soil, poses significant health risks when absorbed by crops and ingested by humans. Therefore, minimizing Cd levels in rice cultivation is an important objective. The α-solanine levels in potatoes were targeted for reduction by suppressing the Solanidine galactosyltransferase (*SGT1*) gene, which catalyzes the conversion of solanidine to α-solanine, using the CRISPRi/dCas9-KRAB tool [282]. This approach aimed to specifically lower α-solanine, a neurotoxic glycoalkaloid, while keeping α-chaconine levels unchanged to ensure the plants’ tolerance to abiotic stress. The developed transgenic potatoes demonstrated reduced α-solanine levels without significant alterations in α-chaconine or nutritional content compared to untransformed plants. In another study, the delivery of RNP into stevia protoplasts resulted in the activation of the UDP-glycosyltransferase 76G1 (*UGT76G1*) gene, which could enhance the production of steviol glycosides used for creating non-caloric natural sweeteners [283].

In terms of plant life cycle, epigenome editing can be utilized to manipulate the timing of critical developmental stages. CRISPR-dCas9 was utilized for targeted DNA demethylation at the FLOWERING WAGENINGEN (*FWA*) locus using TET1cd and a SunTag system that led to late-flowering phenotype upon loss of DNA cytosine methylation [284]. Heritable demethylation through a viral guide RNA delivery system, where the guide RNA was fused to tRNA to facilitate movement within plants was also achieved [285]. Additionally, targeted DNA methylation of the *FWA* and *SUPERMAN* promoters in *A. thaliana* was performed, leading to developmental changes [286]. This involved using the SunTag system to recruit the *Nicotiana tabacum* DRM methyltransferase, with multiple guide RNAs employed to cover the wild-type methylation. The targeted methylation of the *FWA* locus resulted in an early flowering phenotype, as expected. Ghoshal et al. also demonstrated similar targeted methylation effects on the *FWA* locus, using a variant of bacterial CG-specific DNA methyltransferase MQ1 to enhance CG methylation and silencing [287]. The catalytic domains of histone-modifying enzymes G9a, KYP, and human HAT p300 were fused with MS2 to target the FLOWERING LOCUS T (*FT*) gene [288]. While MS2-G9a did not induce late flowering, MS2-KYP transgenic plants showed this phenotype in the next generation, despite no significant increase in H3K9 dimethylation at the *FT* promoter and reduced *FT* expression. Meanwhile, MS2-p300 increased H3K27 acetylation at the *FT* promoter with minimal impact on FT expression and flowering time. These findings suggest that transcriptional regulation does not always correlate with histone modification changes at target genes [289]. The dCas9-JMJ13 tool was designed to manipulate the H3K27me3 mark at the CUP SHAPED COTYLEDON 3 (*CUC3*) developmental gene. The removal of the trimethyl mark on H3K27 resulted in lower growth rates and changes in organ development [290]. The dCas9-VP64-MS2 system was employed to activate the BABY BOOM2 gene (*ZmBBM2*) in maize as a strategic approach to induce parthenogenesis by bypassing fertilization [291]. This gene functions as a developmental checkpoint critical for initiating embryogenesis. In apomictic crop breeding, embryos developing directly from unfertilized egg cells (ECs) allow the fixation and propagation of a stable maternal genotype across generations without genetic recombination. By activating *ZmBBM2* specifically in egg cells in vivo, the system triggers maternal cell-autonomous parthenogenesis, leading to the formation of haploid seeds, demonstrating a significant step toward engineering apomixis for crop improvement. In total, regulation of the life cycle allows for better synchronization with environmental conditions and market demands, ultimately leading to more efficient crop production.

Lastly, enhancing resistance to infections and pathogens is a crucial application of epigenome editing. The CRISPR/dCas9 system has been used to activate the PATHOGENESIS-RELATED GENE 1 (*SlPR-1*) in tomato for defense against *Clavibacter michiganensis* subsp. *michiganensis* [292]. In this approach, dCas9 was fused to the catalytic domain of the tomato gene *SlATX1*, an ortholog of the Arabidopsis histone H3 lysine 4 tri-methyltransferase ATX1 and VP64 activation domain. This fusion induced H3K4 trimethylation and activation of the SlPR-1 locus, enhancing the plant’s defense response. Using CRISPRa, the transcription of the Thioredoxin H (*TrxH*) gene was enhanced, leading to increased resistance to sugarcane mosaic virus (SCMV) in the plants [293]. Elevated expression of *TrxH* conferred this enhanced resistance.

dCas9 constructs have already been used in plants such as Arabidopsis thaliana, *Nicotiana benthamiana* (tobacco), Oryza sativa (rice), Zea mays (maize), *Solanum lycopersicum* (tomato), *Stevia rebaudiana*, *Vitis vinifera* (grape), *Gossypium hirsutum* (cotton), *Triticum aestivum* (wheat) [294], *Solanum tuberosum* (potato), and *Saccharum* spp. (sugarcane) [295]. While epigenetic editing of endogenous genes has been tested, phenotypic effects have often not been described. For instance, editing of *OsER1* and *OsNRT1.1A* in rice, despite their roles in panicle morphogenesis and nitrogen utilization, respectively [296,297], did not yet show clear phenotypic effects [272]. On the other hand, some studies have focused on genes with clear phenotypic effects, such as the *ChlH* gene (magnesium chelatase subunit), which led to yellow seedlings [298], and *PDS* (phytoene desaturase), known for leaf bleaching [299]. However, these genes may lack agrobiotechnological relevance. Future efforts should focus on assessing the phenotypic effects of edited endogenous genes or testing protocols on agriculturally valuable targets. For instance, editing ripening-related genes like PG (polygalacturonase) in tomatoes could optimize harvest times and extend shelf life. Experiments using CRISPR/dCas9 to increase methylation in the promoter region of PG are ongoing [300]. Another promising area is the regulation of non-coding RNAs, such as miR160, involved in leaf and floral development [301]. Although Cas9 has been used for editing ncRNA genes, true epigenetic editing requires leaving DNA intact. Cas13-based systems, developed for RNA editing, show potential, as they have already been applied to modify mRNA and viral RNA in plants, though ncRNA editing remains underexplored [274]. Finally, advancing delivery methods is crucial. Agrobacterium-mediated delivery is limited to certain species, and protoplast-based methods are not universal [248]. Viral vectors, especially with compact systems like Cas12j2, offer promising alternatives for broader applications [272,302]. Additionally, the demonstrated heritability of epialleles (e.g., in the *FWA* gene) and DNA-free RNP delivery methods further highlight the potential of epigenomic editing tools. In the long term, these tools could significantly accelerate plant breeding without altering the genome, potentially classifying them as non-GMO.

The expanding toolkit of epigenome editing in plants, including dCas9-based systems, allows for precise modulation of gene expression without altering genomic sequences. These technologies have demonstrated potential across multiple plant species and traits, providing a powerful platform for advancing sustainable crop development.

### 5.3. Real-World Applications and Regulatory Considerations

Recent progress in epigenome editing has extended beyond proof-of-concept studies to include field-tested and commercially promising applications in major crop species. For example, California-grown tomatoes treated epigenetically remained fresh longer after harvest. Decibel Bio’s platform (a Gates-funded startup) applied an epigenetic “treatment” to tomato seeds (exposing them to small DNA fragments from the plant itself), which “tuned down” natural fruit-degradation enzymes without changing the DNA. Treated fruits were phenotypically identical to controls but resisted post-harvest senescence much longer. This experiment used a non-transgenic seed treatment (possibly dsDNA or sRNA) to trigger durable epigenetic repression of ripening factors, demonstrating a reversible, field-validated epigenome editing approach. Field-scale deployment of epigenetic priming has also been demonstrated in staple crops such as soybean, canola, and sorghum. Epicrop Technologies developed a non-transgenic grafting system that delivers small RNAs from rootstocks to elite scions, inducing beneficial gene expression patterns through heritable epigenetic marks. This method resulted in improved drought and heat tolerance, enhanced germination under cold stress, and increased yield, all without introducing foreign genetic material. Notably, the induced traits persisted across multiple plant generations, underscoring the agricultural potential of non-genomic, heritable epigenetic interventions.

The regulatory status of such crops varies by jurisdiction but generally favors non-transgenic approaches. In the United States, the USDA’s product-based regulatory framework exempts genome-edited plants from GMO oversight if they do not incorporate foreign DNA or plant pest elements. Epigenome-edited plants, which retain their original DNA sequence, are likely to be treated similarly. In the European Union, the 2001/18/EC GMO Directive focuses on genetic sequence alterations, and legal interpretations suggest that purely epigenetic changes—especially those that do not involve transgenes—may fall outside the scope of the regulation. Meanwhile, in China, recent reforms have created a distinct approval pathway for gene-edited crops lacking transgenic elements. Although specific policies for epigenome editing are still emerging, the transgene-free nature of these approaches aligns well with current regulatory preferences in all three regions.

Collectively, these developments underscore the dual advantage of epigenome editing for agriculture: the ability to generate precise, stable phenotypic outcomes and the opportunity to navigate regulatory systems more efficiently than traditional genetically modified organisms. As regulatory bodies increasingly recognize the distinction between genetic and epigenetic modifications, the commercial potential for crops improved through epigenome editing is likely to expand. These technologies not only meet agronomic challenges such as yield stability, stress tolerance, and food quality but also provide a path forward that may be more acceptable to consumers and policymakers concerned with genomic integrity.

## 6. Industrial Biotechnology Application

Industrial biotechnology is the application of biotechnology that uses enzymes or microorganisms for the sustainable, large-scale processing and production of chemicals, materials, enzymes, antibiotics, fuels, and other important products required by society. The goal of industrial biotechnology is to develop sustainable, efficient, and innovative production processes while reducing environmental impacts and transitioning from a fossil-based economy to a bioeconomy [303,304]. Industrial biotechnology mostly relies on using different types of bacteria, yeast, filamentous fungi [305] and microalgae [306]. Bacteria, such as *Escherichia coli*, *Bacillus subtilis* and *Corynebacterium glutamicum* are engineered to produce biofuels, bioplastics, and pharmaceuticals [307,308,309]. Filamentous fungi, such as *Aspergillus niger*, are widely used in enzyme production and organic acid synthesis [310]. Similarly, microalgae like *Chlamydomonas*, *Chlorella* and *Spirulina* are crucial for producing biofuel and high-value compounds like antioxidants and vitamins, while also sequestering CO_2_, reducing greenhouse gas emissions [311,312]. Yeasts, such as *Saccharomyces cerevisiae*, are used in the food and biofuel industries [313].

CRISPR/Cas tools open new possibilities for genome editing and transcriptional control in metabolic engineering [314]. Biosynthesis of metabolites in microorganisms usually depends on the interaction of several enzymes that are encoded by multiple genes. Genome editing using CRISPR technology is a powerful tool to implement new or adjust available metabolic gene networks in microorganisms. However, the low level of efficiency of homologous recombination in many industrial bacterial and fungal species complicates their modification through simultaneous editing at multiple loci. The dCas systems are also being actively explored to regulate gene expression for metabolic bioengineering purposes [314,315] (see Supplementary Table S3 for a list of such applications, also available as an interactive table at https://intbio.org/Kovalev_et_al_2025/ST3, accessed on 24 June 2025). The introduction of dCas-based epigenetic modifiers in microorganisms may be used to bias the expression of specific genes in a favorable direction.

For example, in bacterial systems, CRISPRi, based on steric blocking of RNAP, has been used in *E. coli* to regulate the synthesis pathway of isopentenols, which may serve as biofuel and a precursor for other industrial chemicals [315,316]. Similar technologies in bacteria have shown significant increases in the yield of target products and have been used to increase the production of amino acids [317], fatty alcohols [318], and medical drugs such as N-glucosamines [319], used to treat inflammation, and epothilones [320], potential anticancer agents. Although these studies demonstrated the possibility of using a dCas system to regulate transcription in bacteria and improve the synthesis of commercially important products, the employed molecular mechanisms did not involve changes to the epigenetic markup of the genome per se (dCas acts like a DNA binding transcription factor). Since the bacterial genomes are rather simple compared to eukaryotic ones, epigenetic modifications play a rather limited role. There are no nucleosomes in bacteria and DNA modifications usually play a role as a part of the restriction-modification system [321,322], which is used as a defense mechanism against foreign DNA (predominant DNA methylation type in bacteria is m4C (N4-methylcytosine) and N6-methyladenine (6 mA), in contrast to m5C (5-methylcytosine) in eukaryotes [323]. Yet, it has been shown that DNA modification in bacteria is also involved in the regulation of the transcription and synthesis of secondary metabolites [324]. For instance, in an industrial strain of *Streptomyces roseosporus L30*, it has been demonstrated that methylation of a specific locus may increase damtomycin production [325]. Although there has recently been an increasing number of studies focusing on epigenetic regulation in prokaryotes [326], to our knowledge the CRISPR/dCas systems have not yet been applied to manipulate these mechanisms with industrial biotechnology applications in mind.

The application of dCas-based tools in eukaryotic organisms relevant for industrial biotechnology has also been explored. For example, in the yeast *Yarrowia lipolytica*, it has been shown that two-level regulation based on dCas9 binding and subsequent involvement of transcription factors by secondary metabolites leads to a sustained increase in the application of dCas-based tools in eukaryotic organisms relevant for industrial biotechnology. For example, in the yeast Yarrowia lipolytica, it has been shown that two-level regulation based on dCas9 binding and subsequent involvement of transcription factors by secondary metabolites leads to a sustained increase in flavonoid production that persists up to 324 generations [327]. In this system, the first level carries out negative regulation of fatty acids to suppress lipogenic pathways that compete with the synthesis of the final product. Thus, gRNAs, under the control of fatty acid-inducible promoters, were targeted at lipogenic pathways and inhibited lipid synthesis when they were over-accumulated in cells. Such regulation leads to increased synthesis of naringenin (from Acetyl-CoA), which, in turn, is an inducer of the transcription factor FdeR and activates the promoter of leucine synthesis, which ensures the growth of the host cell auxotrophic for leucine and stimulates cell growth. Control of gene transcription by using dCas9 and dCas12a fused to epigenetic effectors has also been demonstrated in yeast [327,328]. Epigenetic regulation in the *Saccharomyces cerevisiae* has improved yields of industrially important products such as 3-hydroxypropionic acid and triacylglycerols [329,330,331]. In a recent study, dCas9 fused to the repression factor Mix1, which is involved in the recruitment of histone deacetylases [332], was successfully applied to regulate the *IAH1* gene in *Kluyveromyces marxianus DU3*. This yeast strain was isolated in the fermentation of the Mexican beverage mezcal and is involved in the synthesis of esters, which imparts flavor to the beverage [333].

Another industrially important group of organisms is the filamentous fungi of the genus *Aspergillus*. The possibility of epigenetic editing in *Aspergillus niger* to regulate the synthesis of secondary metabolites, such as brevianamide F, fumonisin B2 and melanin, was first demonstrated by fusing dCas9 to various histone-modifying factors [334]. Melanin has potential in industrial applications and has been used to address bioremediation, nutraceutical, and energy challenges [335], and brevianamide F is an antibacterial and antifungal agent [336,337]. In another study, LbdCas12a fused to the VPR activator domain led to an increase in the synthesis of another fungal secondary metabolite, microfuranone [338], whose function is not fully understood.

CRISPRi technology was also applied to increase lipid production in the microalga *Chlamydomonas reinhardtii*. The use of the dCas9-KRAB repressor increased lipid synthesis by 1.9-fold compared to the wild-type strain, which is important for the use of microalgae as biofuel feedstock [339]. Another example of epigenetic control in microalgae was demonstrated in the industrially important species *Chlorella sorokiniana* [340]. Due to the lack of genomic data, the authors used a gRNA consisting of 20 guanines to randomly activate/repress genes, which resulted in an increase in lipid and protein synthesis in cells.

Taken together, dCas-based systems are showing promise in optimizing the metabolism of microorganisms for industrial biotechnology applications. Metabolic engineering often requires simultaneous optimization of the activity of many genes along the metabolic pathway; we envision that more complex gene regulatory circuits that can be implemented using dCas-based systems (review in [341]) may be at some point used to this end.

## 7. Challenges Associated with the Use of Epigenomic Editors and Ways to Overcome Them

Epigenome editing technologies are providing unique tools for manipulation of gene expression and genome functioning. They supplement genome editing technologies and in certain tasks may be used instead of them, while being technically easier to implement. A key feature of epigenome editing is its ability to both silence and activate gene expression. The achievable levels of gene silencing are similar to those achieved through gene knockouts [44]. Moreover, genome editing can lead to indels, deletions, and other genotoxicity consequences due to off-target editing. In contrast, dCas protein-based systems are less prone to off-target effects when targeting single genes and do not exhibit significant side effects when applied in multiplexing scenarios [342]. “On demand” gene activation of endogenous genes is a unique capability provided by epigenome editing technologies. Compared to exogenous gene expression using plasmid transfection, epigenetic activation preserves the native context of the gene, its post-transcriptional processing, it is not limited by the size of the open reading frame and may be used for multiplexed activation of several genes. For many therapeutic applications simultaneous targeting of multiple genes in the genome is necessary. In the case of epigenomic editing, multiplexing may be also used to induce combinatorial synergistic effects and increase the degree of gene repression or activation [343]. As described in the previous sections some diseases are associated with alterations in the epigenetic context, making them immune to gene editing-based therapies. Moreover, epigenetic modifications are reversible, which adds another level of control and flexibility. However, epigenetic editing faces various other challenges, which we discuss in this section.

### 7.1. Challenges in Genome Target Selection (Off-Target Effect) and Context-Dependent Effects

An important problem of dCas-based epigenetic editing is the selection of target sequences for epigenetic effectors. A good sgRNA should have a spacer that targets in a specific way a genomic locus that is optimal for inducing a desired response through epigenetic editing. The presence of off-target effects is one of the most important limitations for use in clinical practice. Off-target effects can be caused by both low specificity of dCas proteins and nonspecificity of epigenetic effector domains. Increased specificity of both dCas proteins and effector domains is achieved through gRNA design and protein engineering techniques. For example, a variant of DNMT3A with a highly specific mutation has been created that reduces off-target effects by reducing DNA binding [344].

While minimizing the off-target effects of dCas proteins can be achieved by designing sgRNAs using a suite of bioinformatics tools used for genome editing [345,346], a bigger problem is the selection of the target locus itself to achieve the proper activity for the epigenome editor. The selection of one specific sgRNA sequence is difficult because usually the targeted promoter regions are rather long and may be located both upstream and downstream of the transcription site. In addition, enhancer sequences may be also targeted to affect the transcription of their respective genes [347,348].

The effectiveness of epigenome editors is influenced by a number of factors, including the DNA sequence of the targeted locus, its epigenetic state, as well as the genetic and epigenetic context surrounding the locus within the 3D structure of the genome in the nucleus [343]. Different cell types are known to have different epigenetic landscapes and gene expression patterns, suggesting that the choice of epigenetic editors should be customized to match the cell type, the specific epigenetic state of the targeted gene and molecular mechanism employed in the maintenance of the epigenetic state. Indeed there is increasing appreciation that factors such as underlying DNA motifs and variants, and the cell type-specific repertoire of TFs, will all modulate the precise impact of a chromatin modification at a given locus. One study compared the effects exerted by epigenetic editors that set different histone PTMs, and found that even actively expressed genes in two cell types with similar epigenetic profiles may manifest very different responses to certain variants of dCas protein-based epigenetic effectors [349]. In this study performed on HEK293 and HCT116 cells, actively transcribed HER2, MYC and *EPCAM* genes were selected for repression by seven different epigenetic editors. As a result, for example, for the *HER2* gene, epigenetic editors fused to Ezh2 and DNMT3A-elicited a significant degree of repression in HCT116 cells, while FOG1 and KRAB domains were more effective in HEK293 cells. Another important result of the work is the finding that editors based on catalytic epigenetic domains are less susceptible to epigenetic contexts.

Often trial-and-error approaches are used to select the optimal genomic locus and the corresponding sgRNA sequence, which is time consuming. High-throughput experimental screening of sgRNA libraries may be used to facilitate sgRNA selection, especially when multiple gRNAs targeting simultaneously different genes are designed [350]. Simultaneous targeting of multiple targets may also increase the persistence of epigenetic edits [351]. Recently, a number of machine learning tools have been developed to predict the activity of different editors targeting various genomic sites [352,353]. Such models try to incorporate information about the epigenetic markup of the specific cell lines or tissues that are considered for editing [354,355]. The use of such tools we were able to predict gRNAs for successful activation of 8 genes using dCas9-p300 [355].

Thus, although the efficacy of epigenetic editing depends on multiple factors including epigenetic context, cell type, effector domain, etc., resulting in different efficiencies in gene activation/repression levels, state-of-the-art approaches are being developed to predict the outcome of the technology for different contexts.

### 7.2. Potency of Epigenetic Editing and Persistence of Epigenetic States

While genetic modifications are maintained during somatic cell division, and successful modifications guarantee their mitotic heritability, the level of persistence of epigenetic states introduced through epigenome editing is an open question. In certain cases it was shown that persistent expression of epigenome editing constructus was needed to maintain that activated or silenced state of the target gene. For instance, repression based on the widely used KRAB repressor domain has been shown not to be long-lasting and requires continuous expression of the effector in somatic cells. The same is true for activator tools of epigenetic editing. For example, several studies have reported the loss of effect when activating gene expression with effectors based on the widely used VP64 domain [356]. Therefore, increasing attention has been paid to the development and optimization of existing epigenome editing tools based on dCas proteins that are capable of inducing long lasting epigenetic memory effect through their transient expression in cells. For example, the CRISPRoff system (dCas9 fused with KRAB-DNMT3A-DNMT3L) [46], the dCas9-system conjugated to KRAB-MeCP2 effectors [48], the CHARM system (dCas9 fused with H3tail-DNMT3l) [49], which combine different domains as shown demonstrate robust long-term gene repression effects. It has also been shown that exposure to EvoETR (evolved engineered transcriptional repressor) when delivered as mRNA LNPs results in effective and long-lasting repression (up to 90%) of the *Pcsk9* gene [357,358]. Among activating epigenetic editors, the recently developed vCD domain in combination with VP16 showed sustained gene activation, which was maintained during somatic cell divisions [55]. Thus, depending on the specific target, and the desired application scenario different epigenetic editors should be chosen.

### 7.3. Delivery Methods

There are many ways to deliver components of the CRISPR/Cas system in the form of DNA, RNA or RNPs. The delivery may be accomplished through viral and non-viral delivery systems. Non-viral approaches can be used to deliver DNA, mRNA, and RPNs and are performed via electroporation, liposome-mediated delivery, microinjection, and in the form of nanoparticles [359]. However, in the case of epigenetic editing, long-term expression of transcription factors is usually required. Therefore, viral delivery methods based on AAVs, lentiviruses or baculoviruses are the most widely used [360]. For tasks related to gene therapies, rAAVs are usually used instead of AAVs. The former lack the most part of the wild-type genome, and cannot integrate into the host genome [361,362]. Also an advantage of rAAV is the availability of a large number of serotypes with a wide range of tropism to different cell types. In addition, there are serotypes of rAAVs that can cross the blood-brain barrier [363], which is important for developing therapies for neurodegenerative diseases. However, the high prevalence of adenovirus infections in humans results in a high level of pre-existing immunity against AAV of different serotypes, which limits their use as therapeutic agents [364]. Another limitation of rAAV is its capacity, which is only about 4.7 kb. This is a major limitation in the case of epigenetic editing since dCas-based constructs are usually large due to combination of the dCas proteins (e.g., SpdCas9 is already around 4 kb) with other effector domains. However, ongoing efforts to develop dCas proteins of smaller size with similar binding strength to that of dSpCas9 may help to overcome these limitations. Thus, recently researchers have developed epigenetic tools based on SadCas9, dCas12e, CjdCas9, FndCas12a and others. Although AAVs are considered a safe delivery system with minimal immunogenicity, it has been shown that systemic treatment with high doses of AAVs can lead to hepatic, renal, cardiac, or pulmonary failure, causing lethal outcomes. Baculoviral and lentiviral vectors are among other viral systems used for delivery of CRISPR-based epigenomic editors. Although lentiviruses have a large capacity, about 12–15 kb, which allows them to encode the full sequence of SpdCas9 together with epigenetic effectors, they are capable of nonspecific integration into the genome, which limits their therapeutic application. The advantage of engineered baculoviruses is the high transduction efficiency (up to 95%) in transducing certain cell types, such as adipogenic stem cells [365], which are promising therapies for bone and cartilage regeneration. However, they are only capable of temporary transgene expression (less than 7 days). Thus, the use of viral vectors is a promising method that allows efficient delivery of epigenetic editors to target cells. The disadvantages of this type of delivery can be avoided by pseudotyping viral vectors to reduce immunogenicity and increase specificity, as well as minimizing the size of the epigenetic editors in the case of using AAV [366].

Among non-viral delivery methods, mRNA delivery in the form of lipid-nanoparticles (LNPs) has gained practical application [358]. This approach reduces immunogenicity induced by the delivery platform and also completely excludes the possibility of genomic integration, unlike viral vectors. The gRNA and mRNA molecules can be packaged either in separate LNPs or tandemly in a single particle. Although mRNA within liposomes is short-lived, it has been shown that such systems can be used for long-term epigenetic repression in vivo [357,358]. This approach has also been used to deliver epigenetic editors for the treatment of HBV, an approach undergoing clinical trials (NCT06671093).

In the case of CRISPR-based genome editing, the direct delivery of Cas-systems as ribonuclear protein (RNP) complexes is considered to be the most efficient and the safest delivery approach at least for ex vivo applications [367,368,369]. Because epigenomic editors include epigenetic domains of non-bacterial origin, the preparation of such RNP-complexes is difficult due to the common use of bacterial expression systems. However, a recent study has shown promise in this direction reporting successful delivery of dCas-based RNP complexes through nucleofection [370]. Such approach also offers advanced possibilities for multiplex applications by combining different sgRNAs within RNP-complexes. We envision that RNP-delivery methods have further potential when combined with epigenetic editors capable of making persistent changes to the epigenetic landscape (such as CRISPR-on/off systems).

Thus, efficient delivery of dCas protein-based epigenetic editors is a key aspect of their application. In works on epigenetic editing in in vitro systems, delivery is most often mediated by transduction or lipofection with plasmids. In in vivo studies on model organisms, the main methods are delivery based on AAV vectors, which provide stable expression of the editor, or delivery in the form of LNPs, which are capable of short-term but effective and safe action on the target. The minimization of epigenetic constructs for AAV packaging and the creation of non-viral delivery platforms are promising directions.

### 7.4. Immunogenicity and Cytotoxicity of dCas-Based Epigenome Editors

The bacterial origin of the CRISPR/Cas systems make them potentially immunogenic. In this regard, dCas-based epigenome editors face similar problems to the Cas-based genome editors. Many studies have confirmed the presence of antibody and T-cell immune responses against Cas proteins [371]. A study performed on canines with Duchenne muscular dystrophy showed Cas9-specific immune responses when delivered through AAV [372]. It is worth noting that the introduction of AAV without a transgene did not cause an immune response in this model.

The only in vivo application of Cas-derived systems in humans has so far been the attempts to treat a 27-year-old patient with Duchenne muscular dystrophy using a custom CRISPR–transactivator therapy consisting of an rAAV serotype 9 vector that contained dSaCas9 fused to VP64 [373] (see Section 3.3). The treatment led to the death of the patient six days after due to an innate immune reaction to a high load of rAAV. No antibodies to the dSaCas9 were detected in the patient; however, the transgene expression was minimal, and there was likely not enough time for the adaptive immune response to be able to develop. The innate immune response to the AVV-based delivery vectors is a known problem and is another challenge for the application of dCas-based therapies delivered through this approach [374].

The epigenetic tools also include epigenetic effector domains and/or transcription factors in their molecular constructs—hence the immunogenicity and toxicity of these moieties should be also considered. Since some effector domains are of viral origin, potential intracellular innate immunity mechanisms triggered by these moieties should also be considered [171]. The toxic effects of effector domains may be present even for the ones naturally present in humans, apparently due to their high expression levels needed for epigenetic editing. Indeed, several studies have reported cytotoxicity of both repressor and, especially, activator epigenetic effector domains [49,67]. For example, the SunTag system consisting of dCas9-GCN4 peptide fusion that attracted p300-CD-scFV constructs has been shown to reduce cell viability [343]. Therefore, some studies have focused on reducing cytotoxic effects [375]. For example, it was shown that the I1417N mutation in the p300 gene significantly reduces the cytotoxicity associated with overexpression of WT p300 while retaining its catalytic activity [375]. Another approach to reduce cytotoxicity related to overexpression of epigenetic factors is to design dCas-based systems that recruit endogenous factors already expressed in cells. For example, unmethylated tail of histone H3 fused to the epigenetic editor dCas9-D3L leads to activation of endogenous DNMT3A without the need to attach a full-length methyltransferase as part of the epigenetic construct [49]. In another study, the dCas9-based construct was supplemented by an R2 RNA loop, which blocked DNA methylation by interfering with the action of cellular DNMT1 methyltransferase—a strategy alternative to the fusion of TET1-like domains to dCas proteins [65].

Thus, the clinical application of epigenetic editors is limited by the immune response to dCas proteins or delivery vectors and the toxicity of some effector domains. Although many approaches to therapies for various diseases based on dCas systems have been developed and tested in vivo to date, the safety of such drugs is not fully understood.

### 7.5. Ethical and Regulatory Landscape for Introduction of dCas-Based Systems into Clinical Practice

The issues associated with the development of safe, effective and specific epigenetic editing technologies, described above, are closely connected with the respective ethical and regulatory challenges of their translation into clinical practice.

While the ethics of gene editing technologies has been discussed extensively, the discourse on ethical issues associated with epigenetic editing technologies has been lagging behind, although a few nice accounts that attempt to summarize the current thinking have been published [376,377,378].

The paramount question is always related to assessing the potential risks of a therapeutic intervention and balancing it with respect to expected benefits for the patients. While the specific risks of epigenetic editing are dependent on many factors (including the molecular composition of the editor, its delivery route, the targeted disease, etc.), the following are usually in focus: (1) the immunogenicity, (2) toxicity, and genotoxicity of the epigenetic editors and their delivery vehicles, (3) the off-target and on-target (e.g., once silencing or activation of the genes near the intended target occurs) effects, (4) the low effectiveness due to instability of epigenetic edits. Apart from immunogenicity, for other risk factors it is generally assumed that, compared to genetic editing, epigenetic editing is expected to have a more favorable safety profile. Since dCas proteins do not have nuclease activity, potential genotoxicity is primarily associated with the delivery vectors (e.g., unintended genomic integration of AVV vectors), while off-target/on-target effects, if they occur, should be less durable since no changes to the DNA sequence are made. However, only specific safety testing and clinical trials can provide definitive answers in each case. For example, AVV episomes can persist in non-dividing cells for years and long-term monitoring of their effects is needed. Given the abovementioned risks, ex vivo epigenetic editing, wherever possible, is assumed to have a better safety profile than in vivo editing.

The potential effects of genetic/epigenetic editing on the germline, resulting in heritable changes that may affect the quality of life of the patient’s offspring, is an often-cited ethical issue. Trans- and intergenerational inheritance in humans is currently a controversial issue; according to current understanding, the vast majority of epigenetic marks (including those on imprinted genes) are erased and reestablished in two waves of epigenetic reprogramming during organism development [379]. Hence, potential long-term direct effects of epigenetic editing on the germline are unlikely.

The ethical questions of equal access, justice and proper use/misuse of epigenetic editing are similar to those discussed for genetic editing. Many epigenetic editing therapies focus on rare diseases; it is expected that the cost of such therapies may be prohibitively expensive in the near future for the majority of the world’s population. The potential use of epigenetic editing not for curing diseases but for enhancing human traits, is another issue requiring proper ethical assessment and regulation. The World Anti-Doping Agency currently bans the use of nucleic acids that may alter gene expression by any mechanism.

Speaking of the regulatory landscape for the introduction of dCas-based editors into clinical practice, we have to admit that the technology is still at the front line of drug development; so far only one single-patient first-in-human clinical trial was approved by the FDA in USA [NCT05514249], and another trial is in progress in New Zealand and Hong Kong [NCT06671093]. Hence, the regulatory landscape is still expected to evolve. However, the regulatory framework is currently in a much better shape than it was some 25–30 years ago when the first gene therapy trials were developed and executed. Those resulted in the tragic death of a patient (Jesse Gelsinger), causing a setback for the gene therapy field and requiring major regulatory and ethical reckoning, and an overhaul of the guidelines by FDA. Currently, the regulation of dCas-based therapeutics by the FDA will fall under a number of guidance documents relevant to gene therapy (e.g., Preclinical Assessment of Investigational Cellular and Gene Therapy Products, FDA-2012-D-1038 [380], Long-Term Follow-up After Administration of Human Gene Therapy Products, FDA-2018-D-2173 [381], Human Gene Therapy Products Incorporating Human Genome Editing [382]). Those mandate robust mechanisms of action, toxicology, immunogenicity, tumorigenicity, biodistribution, and off-target effects studies. While unfortunately the first-in-human FDA-approved trial of a dCas-based editor resulted in patient death, this will hopefully not lead to a setback in the development of other therapies due to the availability of an established regulatory framework for the approval of gene therapy trials [373]. However, this underscores the need for better biomarkers, risk models, and immune monitoring, especially in gene modulation systems. Long-term monitoring of the effects of epigenetic editing is also needed; currently, a 15-year-long follow-up after administration of human gene therapy products is suggested by the FDA.

The potential application of epigenetic editing to the treatment of rare and ultra rare diseases also poses certain regulatory challenges. Preparing data and receiving even compassionate-use IND approvals for performing treatment on a limited number of patients requires a lot of time and resources, limiting the number of diseases/patients that can be treated [383].

## 8. Concluding Remarks, Outlook and Future Directions

Epigenome editing holds significant promise across diverse fields, including animal biotechnology, agrobiotechnology, industrial biotechnology, and particularly in the development of therapeutics for various human diseases. Although the application of CRISPR-based systems to epigenome editing is relatively recent, interest in this area is expanding rapidly, with over 1000 publications to date.

Despite the growing range and applications of epigenetic tools based on catalytically inactive Cas (dCas) proteins, several challenges remain. A major focus is the optimization of genetic constructs for epigenetic editors, particularly through minimizing the size of dCas proteins and effector domains. Substantial efforts are being directed toward enhancing the efficacy of gene activation and repression by improving the design of existing epigenetic domains and identifying optimal combinations of factors that produce durable effects. Additionally, optimization strategies aim to mitigate the cytotoxicity associated with the overexpression of transcription factors fused to dCas proteins. Recent studies demonstrate that such adverse effects can be alleviated by introducing targeted mutations into transcription factor sequences or by employing strategies that recruit endogenous factors.

The tuning and spatio-temporal regulation of the activity and presence of the epigenome editors in the body is another major direction where further research and development may bring fruitful results. While tuning is needed to elicit the therapeutic responses within the respective therapeutic activity windows, it also enables control over cytotoxicity and immunogenicity. Such regulation can be achieved through tuning expression levels, targeted delivery systems, the incorporation of additional regulatory elements within promoter regions, and the usage of chemicals or light to control the activity of the systems.

The expansion of the repertoire of dCas-based systems will likely continue with the incorporation of new effector domains and combination thereof. Epigenetic regulatory networks in human cells employ a vast spectrum of mechanisms. Complex regulatory networks usually employ a combination of advanced mechanisms that combine DNA and histone modifications, ATP-dependent chromatin remodeling, non-coding RNAs, and changes to the 3D-genome architecture. dCas-based tools that tap into these advanced mechanisms are certainly of great interest. For example, they can be used to reorganize the 3D structure of the genome by directed loop formation between the enhancer and promoter regions through artificial binding of dCas9 fused to dimerization domains or gRNA modifications [384,385,386], or by changing the functioning of loop-extrusion complexes by recruiting CTCF transcription factors to certain sites [387,388]. Directed changes to the 3D chromatin structure with the beta-globin locus, may, for example, be used to switch between the expression of adult and fetal hemoglobin [384]. And this will only happen in the cells where this locus is active, suggesting that the use of such advanced epigenetic mechanisms may contribute to spatio-temporal control of epigenome editing.

Beyond the development of tools there is always the need to identify new targets of these tools within the genome whose epigenetic manipulation may provide practically useful results. High-throughput screening experiments based on CRISPRi and CRISPRa technologies provide, in this regard, a very powerful means for interrogating gene function and understanding the complex regulatory networks used by the cells [389,390,391]. New factors affecting cell differentiation, reprogramming, sensitivity or resistance to stress, organism development and metabolite yield can be thus identified and used for epigenetic manipulation.

As the epigenome editing toolkit continues to expand, there is an increasing need for methods that can rapidly and accurately predict the efficacy of new components. Modern machine learning, artificial intelligence (AI), and mathematical modeling approaches offer powerful solutions. Machine learning can streamline and optimize gRNA sequence selection and enable the prediction of gene expression outputs in complex genetic circuits, supporting the development of epigenetic biocomputers based on dCas systems—systems that have, for instance, been used to modulate body weight in mice [392].

Although CRISPR-based platforms currently dominate the field, future expansions may include alternative RNA-guided systems, or even custom-designed proteins that bind specific DNA sequences. The development of such systems may draw inspiration from a number of other RNA-guided DNA binding proteins, such as Argonaute proteins [393], or the recently discovered TIGR-Tas system, characterized by a smaller Tas protein and gRNA compared to SpCas9 [394]. The progress in AI-driven protein design capabilities also holds great potential for advancing epigenome editing technologies through designing new molecular tools with better characteristics than the currently available ones [395,396,397].

## Supplementary Materials

The following supporting information can be downloaded at: https://www.mdpi.com/article/10.3390/ijms26136371/s1.
